# Porous Silicon—A Versatile Host Material

**DOI:** 10.3390/ma3020943

**Published:** 2010-02-03

**Authors:** Petra Granitzer, Klemens Rumpf

**Affiliations:** Institute of Physics, Karl Franzens University Graz, Universitaetsplatz 5, A-8010 Graz, Austria; E-Mail: klemens.rumpf@uni-graz.at

**Keywords:** porous silicon, electrodeposition, nanostructures, nanoparticles

## Abstract

This work reviews the use of porous silicon (PS) as a nanomaterial which is extensively investigated and utilized for various applications, e.g., in the fields of optics, sensor technology and biomedicine. Furthermore the combination of PS with one or more materials which are also nanostructured due to their deposition within the porous matrix is discussed. Such nanocompounds offer a broad avenue of new and interesting properties depending on the kind of involved materials as well as on their morphology. The filling of the pores performed by electroless or electrochemical deposition is described, whereas different morphologies, reaching from micro- to macro pores are utilized as host material which can be self-organized or fabricated by prestructuring. For metal-deposition within the porous structures, both ferromagnetic and non-magnetic metals are used. Emphasis will be put on self-arranged mesoporous silicon, offering a quasi-regular pore arrangement, employed as template for filling with ferromagnetic metals. By varying the deposition parameters the precipitation of the metal structures within the pores can be tuned in geometry and spatial distribution leading to samples with desired magnetic properties. The correlation between morphology and magnetic behaviour of such semiconducting/magnetic systems will be determined. Porous silicon and its combination with a variety of filling materials leads to nanocomposites with specific physical properties caused by the nanometric size and give rise to a multiplicity of potential applications in spintronics, magnetic and magneto-optic devices, nutritional food additives as well as drug delivery.

## 1. Introduction

In today’s nanoscience there is an increasing demand for low-dimensional and nanostructured systems. New physical properties appear, when a structure becomes smaller than a characteristic length scale which is of great interest in basic research but also for applications as nanometric size integrated circuits [1], in optoelectronics [2] and in magnetic materials for magneto-optical devices [3], as perpendicular media for high density data storage [4] as well as in nanostructures used as functionalized sensors in nanobiology [5]. The effects of confinement and proximity affect the interdependence between the physical length scales and the structure size of the patterned materials. The fabrication of nanopatterned materials is widespread in the fields of physics, chemistry and also in biology. A well established technique to produce nanometric structures is lithography used for top-down strategies [6] or bottom-up growth mechanisms [7]. Self-assembled and self-organized structures are also of great interest due to their elementary fabrication processes. A common usage of this technique is the growth of nanoparticles on a substrate by self-assembly [8] but the formation of porous alumina templates or porous silicon matrices [9,10], as well as the fabrication of nanotubes [11,12] are also established.

Silicon is the most dominant material used for microelectronic devices in today’s semiconductor technology. The desire to develop integrable devices with decreasing dimensions results in downscaling by nanostructuring of the base material. Crystalline silicon in its bulk appearance is commonly not taken into consideration as optical, magnetic or biomedical material but nanostructuring leading to a dramatic change of the properties compared to the bulk material is a method to enhance the functionality of silicon in nanotechnology. Light emitting properties occur due to quantum confinement effects and biodegradability as well as bioactivity of nanostructured silicon is observed and applied in many fields of biomedical and pharmaceutical research [13,14]. Due to the dependence of refractive index modification on porosity porous silicon is interesting for optical applications including waveguide technology. The solubility in body fluids dependent on the porosity renders this material an interesting candidate for potential biomedical and pharmaceutical applications.

In general porous silicon (PS) obtained by anodization of a silicon wafer is a versatile material which can display different morphologies by varying the doping density of the wafer as well as the formation parameters. Nanoporous silicon (2–4 nm) generally can be achieved in using p-type as well as n-type silicon of low and moderate doping density, mesoporous silicon with pore-diameters from 5 to 50 nm is generally formed utilizing highly doped silicon as substrate and for macropore (>50 nm up to a few 10 µm) formation usually moderately doped silicon is employed [15]. All these different types of porous silicon are used in basic research studies but are also appropriate for potential applications.

### 1.1. Historical and general remarks on porous silicon

Porous silicon (PS) was first discovered in 1956 by Uhlir [16] during silicon electropolishing experiments. Turner [17] describes the electropolishing regime where the HF concentration at the anode is decreased and dissolution of silicon in the tetravalent form occurs. If the HF concentration is above a critical value a solid anode film is formed. Since then not much attention was paid to this porous silicon layer, but from the 1990s porous silicon has been under extensive investigation after the discovery of the light emitting properties of nanoporous silicon in the visible region by L. Canham, who showed room-temperature photoluminescence of an anodized p-type silicon wafer [18]. Highly porous silicon as well as silicon nanocrystalls emit light in the red, orange, yellow, green and even blue regime when excited with light of higher energy. These light emitting properties have been the subject of great debate and various possible mechanisms for the phenomenon have been proposed. Efficient luminescence in the red, the so called “S-band”, has been attributed to 2D quantum confinement within silicon quantum wires leading to a widening of the band gap [18]. A correlation between the particle size and the luminescence wavelength has been established and it has been shown that a decrease of the particle size leads to a blueshift of the luminescence peak position [19]. The light emission of porous silicon in the blue regime, the fast “F-band”, has been observed in aged PS and has been attributed to direct transitions in Si crystallites of 2–3 nm in size [20]. Further proposed origins of this blue band are, for example, assigned to defects in SiO_2_ [21], thin silicon quantum wires [22], surface states in very small silicon particles [23], siloxene-like cluster [24] or to OH groups adsorbed on structural defects in SiO_2_ [25].

Porous silicon can be achieved by wet or dry etching. The latter is known as reactive ion etching and is very common in today’s microelectronic process technology. Wet chemical etching of silicon can be performed in alkaline solutions which are mainly used for anisotropic etching or in acidic solutions which dissolve silicon in an isotropic way. Addition of oxidizing agents to the electrolytes is a common procedure to enhance the etch rate. In many cases aqueous HF solutions or HF mixed with ethanol are used. A detailed description of chemical etching in various solutions is given in numerous publications, for example [26,27,28].

The formation of porous silicon is generally performed by anodization of a silicon wafer, whereas different types of electrolytic cell configurations are used [29]. In case of additional illumination during the formation process electrolytic double-tank cells are needed.

By using differently doped silicon substrates and varying the electrochemical parameters pore diameters in a wide range between 2 nm up to a few ten microns can be achieved. The morphology of the PS-structures is also influenced by the crystal orientation of the substrate, anodization regime, the configuration of the electrolytic cell as well as the pre- and post-treatment of the sample. The mechanisms to explain the pore growth depend on the length scales of the structure, whereas the most important are the distance between the pores, the radius of the pore tips and the width of the space charge region [27].

In general it is agreed that electronic holes, supplied from the silicon, are required for the dissolution reaction and these holes are depleted in the remaining silicon walls between the pores. If the dimensions are below the Bohr radius of an exciton the holes are depleted independently of the doping density of the silicon wafer due to quantum confinement [30] which leads to the formation of so called micropores. In this case the pore-diameters as well as the distance between the pores are self-adjusted. All in all a real satisfactory model to explain this formation process has not yet been given.

Mesopores occur by etching of n^+^/p^+^ silicon with applied high current densities due to avalanche breakthrough. In general these pores grow in a dendritic manner, whereas due to different growth velocities the main pores are along the (100) direction and the side-pores along (111) direction. A further explanation of pore growth is given by the current burst model which considers the current oscillations in a quantitative way [27]. 

The formation of macropores can be explained by charge transfer across the silicon/electrolyte Schottky barrier, if the non-planar pore interface is considered [31]. The macropore fabrication in n-type silicon, which is so far best known, is obtained by back side illumination to generate holes at the pore tips. This kind of pores are the most consummate ones considering the smoothness of the pore walls as well as the constant diameter along the entire pore depth [27]. Macropores are usually designed by lithography and have been developed in a nearly “perfect” manner by Lehmann *et al*. [32]. Quite a number of different formation procedures in using aqueous, non-aqueous or organic electrolytes and different types of illumination during the anodization as well as some corresponding growth models are described in detail in literature [33,34,35,36] by various groups.

### 1.2. Functionalization of porous silicon

Porous silicon a versatile material with its various morphological natures is compatible to today’s microtechnology and is of interest for a great variety of applications. The high surface area of this material (nanoporous silicon ~1,000 m^2^/cm^3^, mesoporous silicon ~100 m^2^/cm^3^, macroporous silicon~1 m^2^/cm^3^) makes it suitable to fill the pores with one or more guest materials which results in a drastic change of the physical properties. There have been efforts to exploit this behaviour efforts to build chemical or gas sensors. The detection for example is based on changes of the refractive index, the work function, dielectric constant, resistivity or the light emission caused by distinct chemical materials filled into the pores. Molecules that react with the inner surface of the porous silicon matrix can quench the luminescence by enhancing the non-radiative recombination processes [37]. Furthermore porous silicon, due to its low toxicity, is an interesting candidate for biomedical applications and therefore is used for loading with a payload for therapeutics or diagnostics. This can be achieved by covalent attachment, physical trapping and adsorption [38], whereas biomolecules or drug molecules can be linked to the inner pore walls by covalent attachment. Trapping of nanoparticles (e.g., Fe_3_O_4_) by oxidation of the porous silicon matrix is realized by the shrinking of the pore-diameters due to the oxidation of the pore walls and inclosing the material within the pores. Due to the modification of the porous silicon surface for example by oxidation its nature can change from hydrophobic or hydrophilic. SiO_2_ offers a negative surface charge and thus coulomb forces can attract positive ions [38,39]. This means that the nature of surface chemistry can affect the strength of attachment of a molecule to the porous silicon surface.

The biodegradability of nanostructured silicon renders this material interesting for medical applications such as controlled drug delivery, embedding of specific nutrients in functional food [40] and orthopedics [41]. On the one hand nanostructured porous silicon tablets are utilized to carry a cocktail of drugs or nutrients which will be delivered in a predetermined time within the body and on the other hand percutaneous implants of porous silicon with radioactive content can provide radiation to tumor cells [38].

Furthermore composite materials are formed by impregnation of metals and conductive polymers into the pores, which started as an attempt to improve the electrical contact in electroluminescent devices but is also used to influence the efficiency of the electroluminescence [42]. In most cases the metal deposition results in quenching of the light emission and only a few reports concern affecting the luminescence [43]. Recently the filling of porous silicon structures with metals was investigated with respect to applications for electronic devices [44] but by using ferromagnetic materials [45] devices related to the magnetic behaviour of the composite system are also under investigation. The methods of deposition are either electrochemical, electroless or by impregnation. The use depends on the material and the desired result (complete filling, layer deposition, *etc*.).

### 1.3. Applications of porous silicon

**Figure 1:**
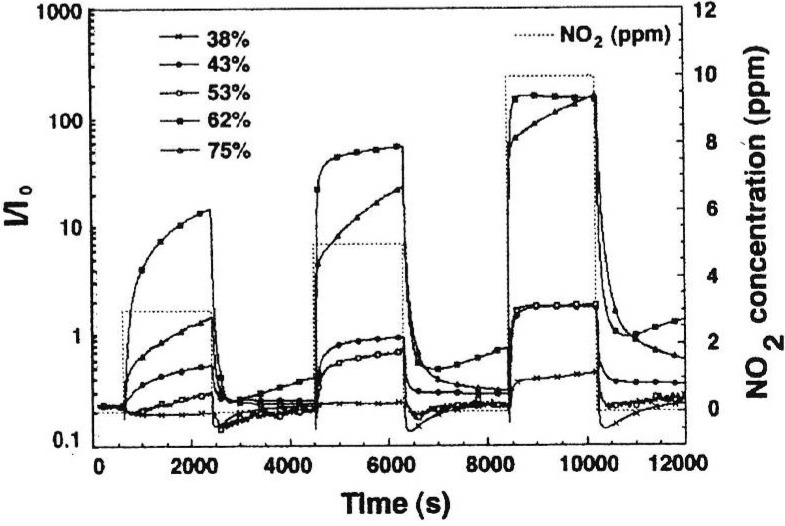
Dynamic response of mesoporous silicon samples of different porosity, varying between 38% and 75% to NO_2_ (diagram taken from [47]).

Porous silicon is a potential candidate for applications in various fields to fabricate on the one hand miniaturized and on the other hand cheap devices. One of the main application areas of porous silicon is sensor technology, whereas the classification of the sensors depends on the parameters which are measured. A commercially fabricated sensor is the HF-tester, which identifies hydrofluoric acid content in a liquid based on the property that an oxide layer formed on silicon is removed by HF. Porous silicon gas sensors are mainly sensitive to the change of the refractive index, conductivity or capacity. Adsorbed polar molecules change the surface charge which leads to a modification of the resistivity or capacity [26,46,47]. Figure 1 [47] shows the dynamic response of porous silicon with porosities between 38% and 75% to NO_2_.

**Figure 2:**
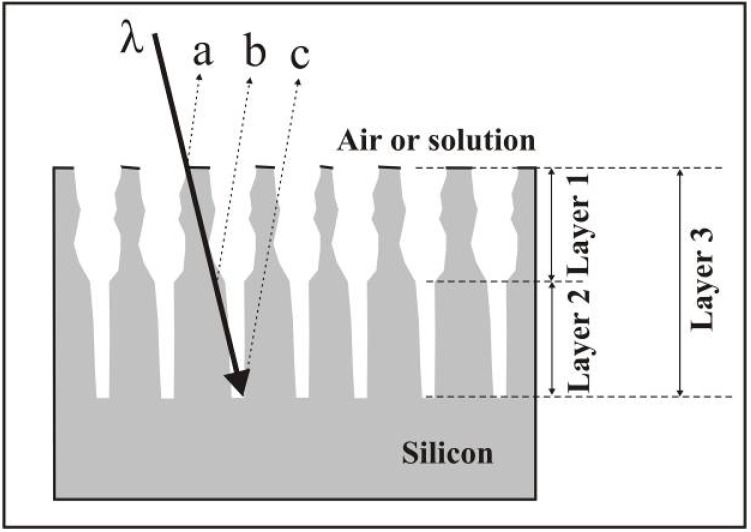
Scheme of a porous silicon double-layer with two different pore-diameters employed as bio-sensor which detects the molecules dependent on their size (after [48]).

Sensing of biomolecules based on changes of the refractive index and the concomitant shift of the spectral position of the Fabry-Perot fringes is described by [48]. A double layer porous silicon structure with two different porosities is used to obtain interference from beams reflected at the interfaces between different porosities. Incorporation of biomolecules leads to the change of the refractive index and thus a modification of the effective optical thickness. Due to this behaviour, molecules of different size can be distinguished. The two layers of the porous silicon structure exhibit different pore-diameters, the lower one a smaller average diameter than the upper one, which means that molecules above a certain size are embedded only in the upper layer and can not penetrate the lower one (see Figure 2, after reference [48]).

The detection of a great number of gas- or biomolecule sensors is based on the change of the optical parameters and is performed by optical measurements. The quenching of the photoluminescence caused by the reaction of reactive species (e.g., iodine, bromine, chlorine) with the porous silicon surface due to surface traps is also utilized [49]. Furthermore quenching of the luminescence by physisorbed molecules is observed (e.g., hexane, benzene) which is recovered if the solvent is removed [50].

**Figure 3:**
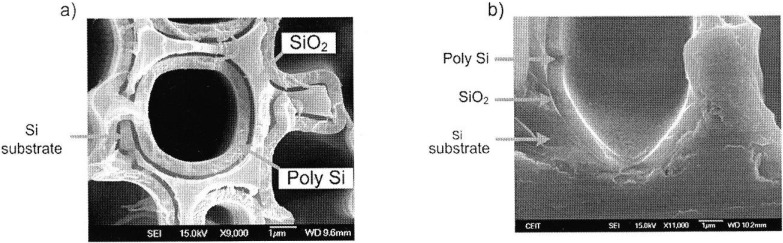
Scanning electron micrographs showing (a) a plan view macropore covered by a SiO_2_ layer and a deposited polysilicon layer acting as top electrode and (b) the corresponding cross-sectional view (image taken from [51]).

Macroporous silicon with a dielectric layer (ONO) covering the pore walls has first been introduced by Lehmann [46] to utilize this material as capacitor. A further kind of macroporous silicon capacitor [51] consists of doped silicon, SiO_2_ acting as insulating layer covering the pore walls and a doped polysilicon layer which forms the top electrode (Figure 3). An increase of the capacity proportional to the enlarged surface is the consequence.

Another promising application of porous silicon to reduce the product costs is its utilization in the fabrication of solar cells [52,53]. Due to the biodegradability and biocompatibility nanostructured silicon is also investigated in relation with its biomedical applicability, for example, in tissue engineering [54] and cancer therapy [55].

## 2. Fabrication of Porous Silicon by Anodic Dissolution of Bulk Crystalline Silicon

One way to achieve nanostructured materials is pre-patterning of a substrate (top-down, bottom-up) but also assembling structures by self-organization is a favourable technique to realize particle growth on surfaces [56] or porous materials [57,58,59] with more or less regular arrangements. In common, systems tend to reach an optimum state with minimal interaction and a minimum of dissipation (minimal waste of energy) [60].

**Figure 4:**
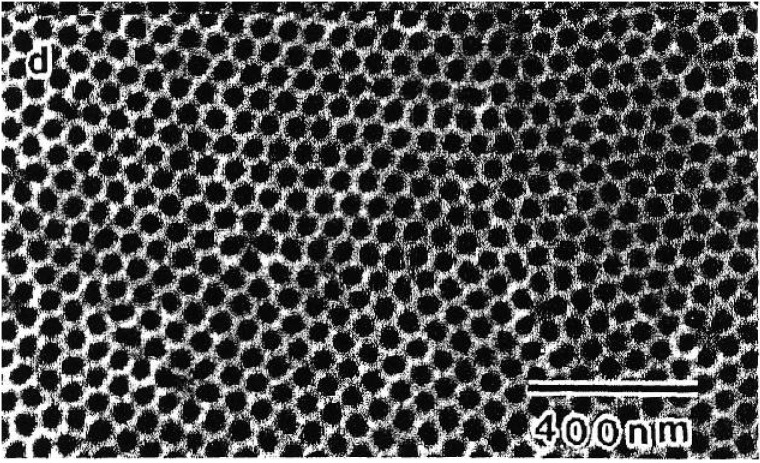
Self-ordered porous alumina template formed under 27 V anodizing potential. Image taken from (reproduced by permission of The Electrochemical Society [61]).

In recent years porous alumina templates which are fabricated by etching of an alumina foil in an oxalic or sulfuric acid solution leading to quite regular close packed honey-comb like pore structures [61], grown by self-organization are used as templates. The regularity of the pore arrangement can be improved by controlling the anodization conditions [62] which results in large defect free regions (Figure 4).

Porous silicon, which can also be fabricated with straight nanoholes by self-organization, offers the possibility to utilize this material as template as well. Recently porous anodic alumina was used for pre-patterning of the silicon surface to promote the formation of ordered porous silicon structures [63,64]. Generally porous silicon is formed by anodization of a silicon wafer in an HF-containing electrolyte. The formation mechanism of porous silicon, which up to now has not been completely and satisfactory understood, is described in various models in relation to the different morphologies. A theoretical description of the pore nucleation in relation to the parameters as dopant concentration, electrolyte concentration, current density, applied potential, illumination intensity, surface energy as well as in considering the hole-transport in the silicon wafer and the ion-transport in the diffusion layer is given by [65]. As a result, a concomitant distance between the pores should be obtained for specific experimentally determined parameters. A quantitative estimation of the pore morphology can be gained from the model by Beale *et al*. [66] which implies the occurrence of a Schottky barrier between the silicon/electrolyte interface due to surface states, current transport through the barrier by electron tunneling (heavy doped silicon) or Schottky emission (lower doped silicon) and the preferential dissolution of silicon at the pore tips caused by the enhanced field strength there. As a result the height of the Schottky barrier is lowered. Other models, such as the one based on the distribution of the electric field at the pore tips [67] or another assuming a quantum confinement effect [68] or some combination of pore nucleation and subsequent growth [69,70] also cannot give a satisfying description of porous silicon formation with its numerous features.

### 2.1. Self-organized pore growth

The pore growth of porous silicon considering the self-organizing nature has been investigated by various groups, theoretically [71,72,73] but also experimentally, for example by using the current burst model [74]. The dynamics of self-organization forming hexagonal arrangements have been shown by [75] in using a defect-deformation mechanism [76]. This model estimates that the pore-distance in silicon is in consistence with the width of the space charge region. A correlation between space charge region and pore-distance has also been found by Lehmann *et al*. [26,77]. A relation between pore-distance (d) and the concentration of mobile carriers (n) is given by *d(n) ~n^-1/2^* [78] taking into account that the etching is stochastic and that the next pore can be formed at any place of the surface more probable in a distance greater than the Debey length (R_D_). These estimations lead to pore arrangements with distances between adjacent pores in the order of the Debey length. These findings are in good agreement with the fabricated porous silicon structures.

Another suitable way to explain the self-assembly of PS-structures seems to follow the assumption of Lehmann that the formation of larger pores (meso/macroporous regime) is caused by a self-organization process, namely Ostwald ripening which is well known in connection with the self-organization of particles or clusters on surfaces. Ostwald ripening is due to the minimization of the free energy associated with the interface of two phases [79]. A theory consistent with these findings has been developed by Lifshitz, Sylozov and Wagner describing cluster size distributions [80]. Porous silicon formation starts with the nucleation of etch pits which is a randomly driven process, as already mentioned above. These stochastically distributed voids lead to growth of a microporous layer. Depending on the anodization conditions like current density, electrolyte concentration and temperature the pores exhibit different size. The growth mechanism of the pores occurs in the way that larger pores grow on the expense of smaller ones due to the energetically favourable ratio of pores to remaining silicon. The major part of the free energy consists of the surface energies of boundaries between the nucleation centres and the matrix. The free energy is lowered in enhancing the small pores to larger ones due to the decrease in the total volume.

### 2.2. Fabrication of quasi-regular arranged mesopores

**Figure 5:**
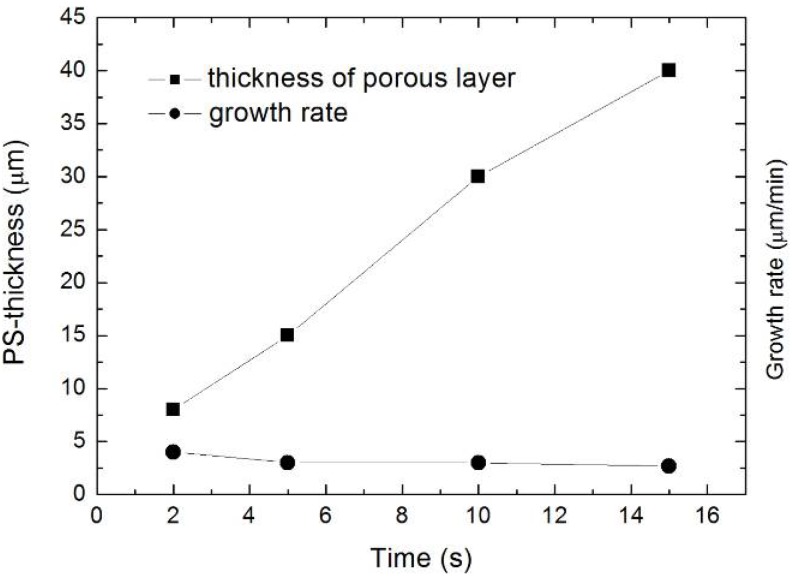
Diagram corresponding to Table 1, showing the parameters thickness of the porous layer and the growth rate in dependence on the time.

During the formation of mesopores with pore-diameters between 45 nm and 95 nm, quasi regular pore arrangements can be achieved. Due to the high doping density of the used silicon substrate (10^19^ cm^-3^) the anodization takes place under electrical breakdown conditions [81] thus the formation process can take place without illumination. The thickness of the porous silicon layer increases monotonically with an enhancement of the etching time (Table 1, Figure 5). Exceeding a certain time (15 min) under the mentioned conditions the electrolyte becomes exhausted within the pores especially at the pore-tips due to the high aspect ratio of the pores (~1,000) and thus no further silicon dissolution which means no pore-growth will occur in these regions resulting in a self-limitation [82] of the etching process. With continuing etching the thickness of the PS-layer is even decreasing because the surface of the sample is attacked by the electrolyte accompanied by dissolution of the PS top layer.

**Table 1:** Relation between anodization time and achieved thickness of the PS-layer. The growth rate drops with increasing etching-time.

Time (s)	PS-thickness (µm)	Growth rate (µm/min)
2	8	4
5	15	3
10	30	3
15	40	2.7

**Figure 6:**
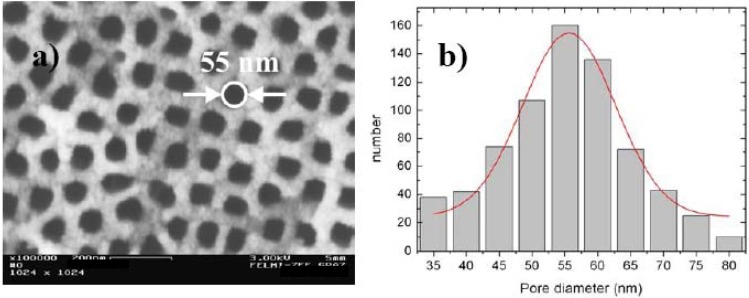
(a) Top view of a porous silicon sample with an average pore-diameter of 55 nm. (b) Pore-size distribution showing the deviation from the average pore-diameter being about 10%.

**Figure 7:**
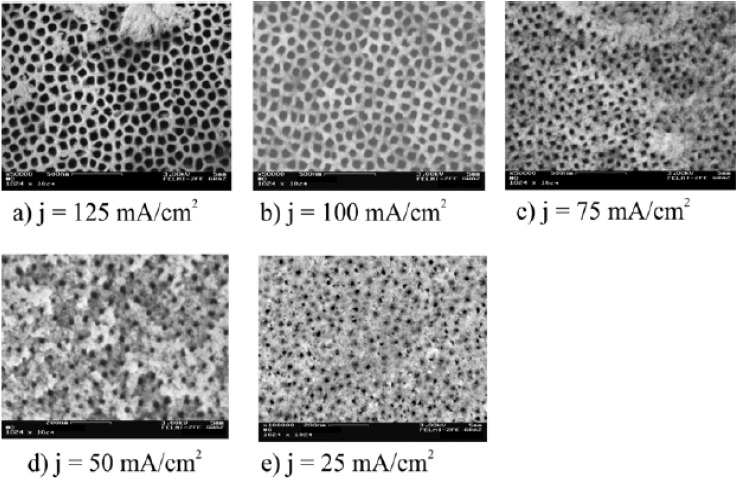
Morphology of the porous silicon template varied by the applied current density [83]. As starting material a (100) n^+^-Si wafer is used. (a) Pore-diameter ~95 nm ±10 nm, pore-distance ~45 nm; (b) Pore-diameter ~60 nm ±8 nm, pore-distance ~50 nm; (c) Pore-diameter ~45 nm ±10 nm, pore-distance ~55 nm; (d) Pore-diameter ~25 nm, pore-distance ~60 nm; (e) random pore size distribution.

**Figure 8:**
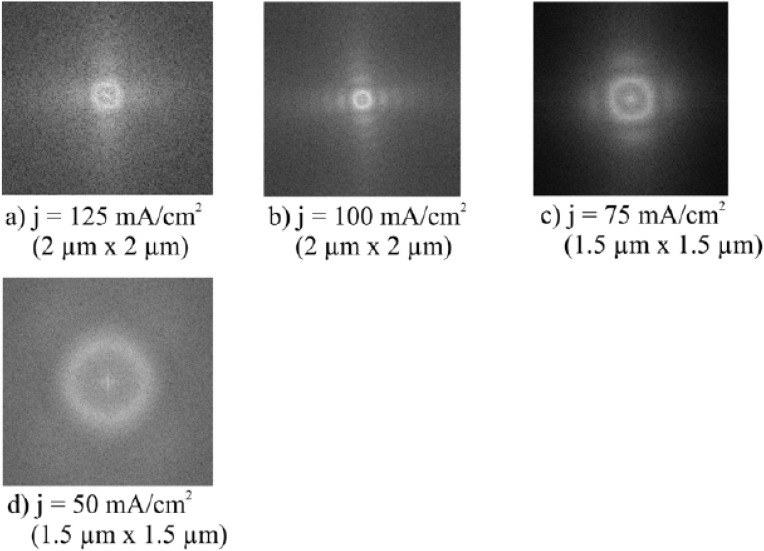
Fourier Transform images corresponding to the top view pictures of Figure 3a–d (images a and b are taken from [83]. The four fold symmetry of the self-assembled pore arrangement can be recognized. The regularity deceases with decreasing pore-diameter and vanishes below a certain value.

Considering one PS-template with a certain pore-diameter (top view shown in Figure 6a) the deviation from the average value of the pore-diameter is about 10% (Figure 6b). Furthermore the pores are arranged in a four-fold symmetry, evidenced by Fourier Transform images of the top view of the samples (Figure 7 and Figure 8) by image processing. To obtain a self-assembled quadratic-like pattern depends on the crystal orientation of the substrate, the doping level but also the anodization conditions as current density, electrolyte composition and concentration as well as on the geometrical arrangement of the electrolytic cell.

**Figure 9:**
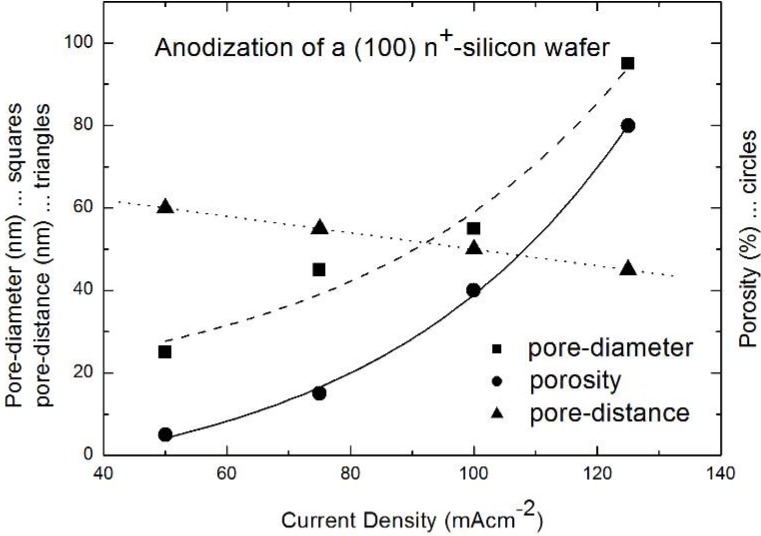
Decreasing pore-diameter and porosity with decreasing current density. The pore-distance is enhanced with decreasing current density (data from [84]).

By increasing the current density from 50 mA/cm^2^ up to 125 mA/cm^2^ the average pore-diameter enlarges from 25 nm to 100 nm. Furthermore the porosity increases too, which means that the pore distance decreases. A relation between current density, pore-diameter, porosity and interpore-spacing is shown in Figure 9. During these experiments in varying the morphology the HF-concentration is kept constant at 10 wt% and always the same doping density of the wafer (n-type Si, 10^19^ cm^-3^) has been used. This means a restriction of the range of variation of the morphology to the mentioned values. The relation between current density and pore-diameter as well as porosity, respectively shows an exponential decay. The pore-distance versus current density follows a linear relation [83].

### 2.3. Pore formation dependent on the crystal orientation

The growth mechanism strongly depends on the crystal orientation of the substrate and is about three times faster in (100)-direction than for the (111) route [85]. To achieve oriented pores separated from each another the pore distance *i* has to fulfill the condition *i ≤ 2⋅SCR* where SCR means the width of the space charge region in the semiconductor. In case of a heavily doped wafer assuming an applied voltage of 1 V the width of the SCR is in the range of 10 nm [86]. The quasi-regular pore arrangement diminishes with increasing pore-distance, accompanied by a reduction of the pore-diameter. Below a certain pore-diameter (~30 nm) the quadratic-like short range order cannot be observed anymore, whereas the channels are randomly arranged. The self-formation process of porous silicon leading to a variety of morphologies with structure sizes between a few nanometers ranging up to tens of microns is still not completely clarified, especially the quasi-regular pore-growth is still non-understood.

The regularity of the PS-template strongly depends on the current density, electrolyte concentration as well as the used configuration of the etching cell. The variation of the morphology by the current density is illustrated in Figure 7 (a, b, c, d, e). Figure 8 shows the corresponding Fourier Transform images of the top view of the samples. It can be seen that the regularity is best for high current densities which means greater pore-diameter than pore-distance and vanishes by falling below a certain pore-diameter of about 30 nm. The better regular pore-arrangement with greater pore-diameter can be understood by the higher current density which results in a higher electric field-strength at the pore-tips.

**Figure 10:**
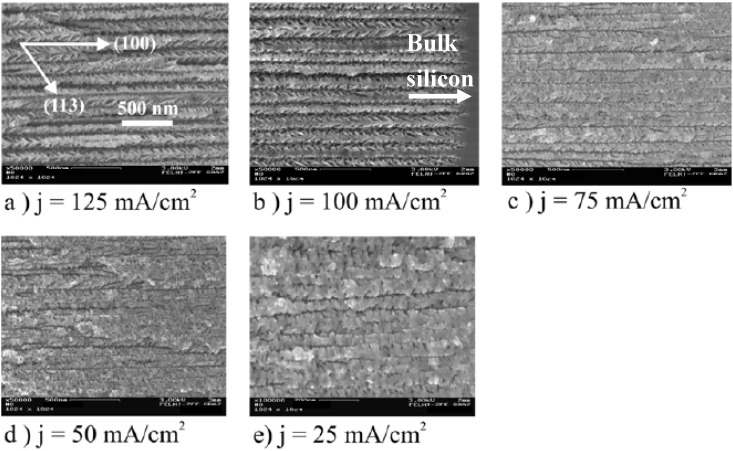
Corresponding cross-sections of the images Figure 3a–e [83]. The pore-diameter decreases from ~95 nm to ~25 nm. Images a, c, d, e show cross sectional views in the mid of the porous layer. In image (b) a region near the pore tips is shown.

Regarding the corresponding cross-section images (Figure 10) of the investigated samples one can see that the growth of side-pores depends on the pore-diameter especially on the growing pore-distance. The increasing pore-distance with decreasing pore-diameter leads to an enhancement of the length of the dendritic grown side-pores.

In case of a pore-diameter between 55 nm and 100 nm the length of the side-pores do not exceed the values of the pore-radius but with increasing pore-distance (pore-diameter between 20 nm and 40 nm) the length of the dendrites increases up to about five times of the pore-radius. This behaviour can be explained by the fact that the interpore spacing exceeds the necessary relation between pore-distance and space charge region being equal or smaller than *2>⋅SCR*.

**Figure 11:**
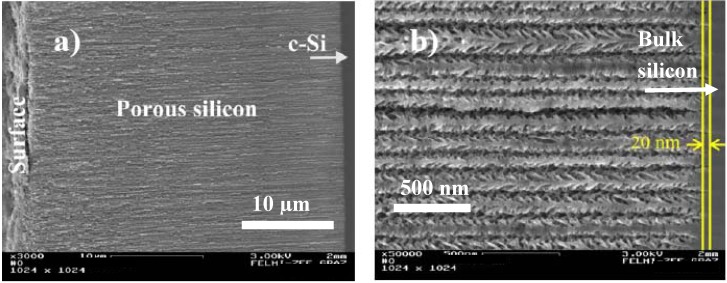
(a) SEM-image of the entire porous layer of 35 µm, (b) shows the pore-tip region in detail exhibiting a deviation of the pore-length of about 20 nm.

Furthermore one can see in looking at the SEM-micrographs that the main pores are growing in (100)-direction whereas the side-pores preferentially offer the (113)-direction with an angle between main-pores and dendites of 54° which corresponds to the crystal structure of silicon meaning the angle between the (100) and (113) direction. The termination of the pore-tips is very abrupt and thus the interface between porous layer and bulk silicon offers only a roughness of about 20 nm (Figure 11b). Compared to the entire thickness of the porous layer of 35 µm (Figure 11a) the variation is much less than 1%.

By varying the electrolyte concentration the pore-diameter of porous silicon structures can also be modified, especially their interpore-distance (Table 2). In keeping the current density constant the pore-diameter decreases and the pore-distance increases with an enhancement of the HF-concentration which means that the porosity decreases which has been observed by other groups too [87,88]. This behaviour is clarified due to the fact that the HF-concentration *c* is related to the critical current density *J_PS_* (equation 1) which is present at the pore-tips (valid under the condition that the current density is limited by the hole generation) [26]:

(1) $$J_{PS}=Cc^{3/2}\text{exp}(-E_{a}/kT)$$

with C = 3300 A/cm^2^, c … in wt % HF, E_a_ = 0.345 eV, T … temperature.

The critical current density is indirect proportional to the pore-diameter ( $d=i{\left(\frac{J}{J_{PS}}\right)}^{1/2}$ ) and thus an enhancement of the HF-concentration leads to a decrease of the porosity.

A quasi-regular pore-arrangement as shown in Figure 7 and Figure 8 can only be achieved in a small parameter regime. The variation of the HF-concentration has to be smaller than 5wt% if the current density is kept constant to achieve a certain regularity with varying porosity. A greater variation results in more randomly distributed pores and even network-like structures in case of *Δc* >5 wt % HF.

**Table 2:** PS-morphology parameters in dependence of the electrolyte concentration. As substrate a (100) n^+^-Si wafer is used.

Current density [mAcm^-2^]	Electrolyte concentration [wt%]	Pore-diameter [nm]	Pore-distance [nm]
100	15	25	80
100	10	55	50
100	5	Branched pores	-
50	5	95	45
50	3	105	25

Considering Fourier Transform (FT)-images (Figure 8) of the SEM top view of the distinct quasi-regular pore-patterns achieved by variation of the applied current density (Figure 7) show a pore-diameter dependent regularity. A clear four-fold symmetry obtained with high current densities *j* vanishes more and more with decreasing *j* resulting in a random pore-arrangement by going below a critical pore-diameter (Figure 8) which is experimentally found as 40 nm.

### 2.4. Further specific methods of pore formation

A sophisticated method to enhance the regularity of the pores by self-organization, especially the smoothness of the walls of high aspect ratio mesopores is the application of an external magnetic field during anodization [89,90]. Two effects which influence the pore growth are observed during the magnetic field assisted anodization. First the pore-diameters decrease with increasing magnetic field (0–4 T) and second the dendritic growth of the pores is suppressed. As a result the aspect ratio of the pores is enhanced and the uniformity of the pores is increased. The influence of the magnetic field on the anodization process is explained by a correspondence of the pore-tip radius and the cyclotron radius confining the holes which decreases with enhanced magnetic field. Therefore an effective confinement of electronic holes at the pore-tips results.

Porous silicon fabricated as layered structure with different porosities can be formed either by modifying the anodization current density or in using periodically doped silicon substrates. Porous silicon superlattices are used for waveguiding [91] due to the varying refractive indices caused by the change of the porosity. Furthermore these structures are employed in sensor technology, for example for sensing different sizes of biomolecules as described in Section 1.3.

**Figure 12:**
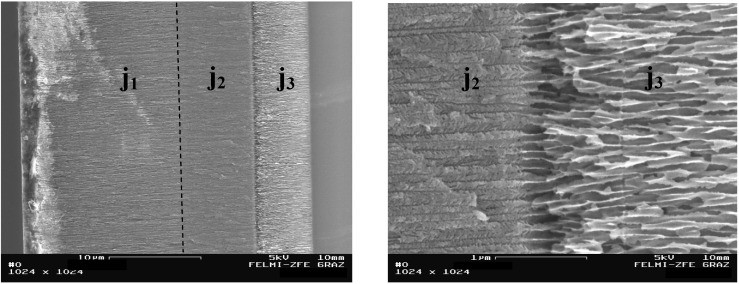
Left: Transition region between three porous silicon layers of different porosities, etched with three changing current densities (j_1_ = 75 mA/cm^2^, j_2_ = 50 mA/cm^2^, j_3_ = 125 mA/cm^2^) . In the right micrograph the zoomed-in boundary between the two layers etched with j_2_ and j_3_ which is quite sharp, is shown. The uncertainty concerning the roughness of the transition lies far below 100 nm.

Figure 12 shows such an extended porous structure with three different porosities etched with three different current densities leading to average pore-diameters of 40 nm, 25 nm and 105 nm, respectively. Achieving layered structures is possible because of the dissolution reaction occurs preferentially at the pore tips and leaves the already formed porous silicon more or less unaffected.

To achieve ordered porous silicon structures patterning of the silicon surface is performed. On the one hand for example porous anodic alumina is used as mask [63,64] as mentioned above and on the other hand the silicon wafer is pre-structured by lithography to realize a regular arranged “lattice” of pores. Especially macroporous silicon is often fabricated by pre-patterning of the wafer. Generally first a mask is produced by photolithography and subsequently the sample is anisotropically etched in a KOH-solution to achieve etch-pits, which have the form of inverted pyramids. In a following step the sample is anodization in a HF containing electrolyte, whereas etching starts selectively at these pits. As a result perfectly grown structures, exhibiting pore-diameters in the micrometer range are obtained [26], which are applied, for example, as photonic crystals [92]. Another kind of preparation is the etching of prepatterned (110) wafers in aqueous KOH solution to fabricate grooved silicon structures which are employed as one-dimensional photonic crystals meaning a refractive index change in the vertical direction [93]. A modification of the lattice period allows the design of 1D photonic crystal with gaps in user-specified wavelength ranges.

## 3. Double-Sided Porous Silicon Samples

**Figure 13:**
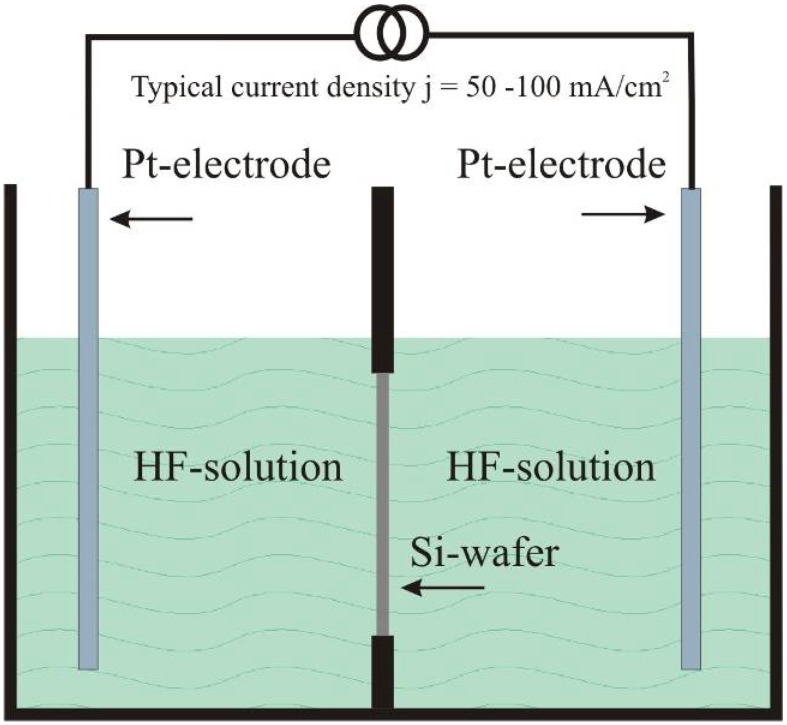
Scheme of a electrolytic double-tank cell to fabricate double-sided porous silicon samples. During the anodization process which is performed under pulsed conditions each side is contacted by the electrolyte. A typically used frequency is 0.1 Hz.

**Figure 14:**
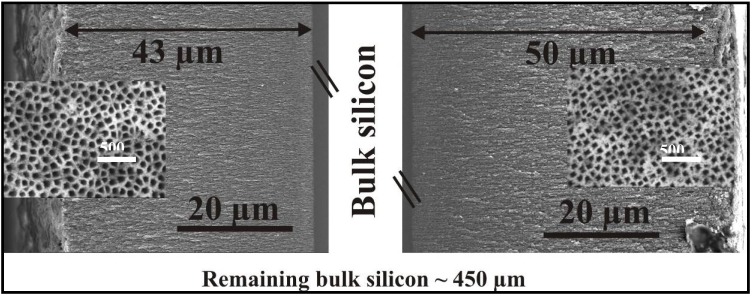
Cross-sectional and corresponding plan view of the surfaces (inset) of the two porous layers.

The fabrication of double-sided porous silicon samples is carried out in a double tank electrolytic cell (Figure 13) where both sides of the wafer are in contact to the electrolyte. The anodization has been performed in one step in applying a pulsed current. The frequency of the anodization current was typically 0.1 Hz [94]. The resulting double-sided samples offer two layers of porous silicon with equal morphology. The pore-arrangement as well as the thickness of the two layers are nearly the same and can be fabricated reproducible. Layer-thicknesses of about 50 µm can be achieved without agitation of the electrolyte. The etching process is self-limiting due to a lack of fluorine-ions within the pores. Figure 14 shows the two porous layers in cross-section as well as the top view images as inset.

### Ultrathin wafers employed for double sided etching

**Figure 15:**
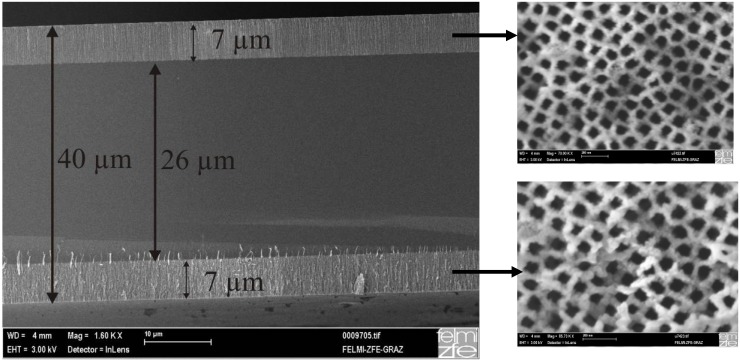
Ultrathin wafer with an entire thickness of 40 µm etched on both sides. The two layers exhibit a thickness of about 7 µm each. On the right side the corresponding top view images show that the morphology is alike for both sides.

In an analogous way to double-sided samples fabricated from a usual silicon wafer, ultrathin wafers are employed to produce single- as well as double sided samples with very thin remaining bulk silicon. First a usual wafer (thickness of 0.5 mm) is thinned in a HF/HNO_3_ solution of about 40 µm–60 µm (sample supply from Intrinsic Materials, Malvern, UK). Then these samples are anodized either single- or double-sided. So far the thickest porous layers which could be etched are about 40 µm. In case of double-samples porous layers of about 15 µm–20 µm on each side could be reached. Figure 15 shows two layers with a thickness of about 7 µm and about 26 µm remaining silicon.

The aim in producing such double-sided samples is to etch the two layers as far as possible with a very thin remaining bulk silicon layer. The closest reachable distance should be twice the width of the space charge region (SCR) where silicon dissolution stops because of a lack of electronic holes. Silicon etching takes place as far as free charge carriers are available. This estimation is also valid for the pore etching as mentioned above in Section 2. Thus it should be possible to achieve a distance of a few tens of nanometers in case of highly doped silicon.

## 4. Filling of Porous Silicon

Beside porous silicon matrices other porosified materials are also used as templates for filling with various materials. Porous structures such as anodic alumina templates, etched polycarbonate foils as well as porous silicon matrices are filled with a variety of different materials, depending on the aim of investigations or applications. Porous alumina structures are mainly filled with metals, especially ferromagnetic ones to investigate the magnetic behaviour and to achieve a perpendicular medium appropriate for high density magnetic data storage [95]. Porous silicon with its numerous morphologies is also filled with metals, conducting polymers but also with molecules to realize gas- or biosensors. In the following the metal deposition within the pores of PS is considered.

### Metal deposition within porous silicon

Herino investigated the deposition of metals and other conducting materials in relation to their influence on the luminescent properties and also due to their modification of the conductivity of porous silicon [42]. The incorporation has been carried out by electrochemical or electroless methods but also by evaporation. Galvanostatic and electroless deposition of Au, Ni and Cu has been performed and it has been shown that electroless metal precipitation is accompanied by an oxidation of the porous silicon surface whereas cathodic deposition does not generate oxide formation at the pore surface [96]. A gradient of the metal content towards the pore tips has been observed. Fe-deposition into the pores, carried out to achieve electrical contact to porous silicon, starts with the nucleation at the pore bottom because of the cathodic standard potential of Fe that is close to the one of silicon [97]. An increase of the electroluminescence has been observed after the deposition of In and Al into porous silicon. The deposition occurs mainly at the pore bottom, which has been explained by the current flow in analogy to the formation of porous silicon. Furthermore deposited metal has been observed near the sample surface which indicates a current path through the nanoporous layer [98]. Silicon/metal and silicon/SiO_2_/metal composites have been fabricated by Jeske *at al*. to achieve binary and ternary nanostructures without and with insulating layers, respectively [99]. The filling of macroporous silicon with Cu by electrodeposition has been investigated by the group of Föll [100], by Fukami *et al*. [101] and other groups [44,102,103]. Föll *et al*. reported the successful complete filling of macropores exhibiting an aspect ratio of 75 (pore-diameter 2 µm, pore-length 150 µm). The porous silicon templates have been post-treated by sonication in CuSO_4_-solution and subsequently dipped in Piranha solution leading to a quite thick oxide layer of about 10 nm. Electrodeposition processes in relation to doped semiconductor materials is not very well understood so far but it can be said that due to the higher field strength at the pore-tips and a concomitant dielectric breakdown of the oxide layer the deposition starts at the pore-bottom. During the deposition the Cu-salt electrolyte was bubbled with N_2_ to remove the oxygen in the solution [100].

**Figure 16:**
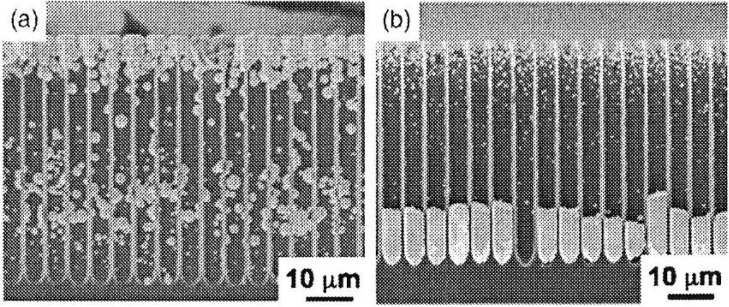
SEM micrographs of cross sectional regions of metal filled macroporous silicon. (a) Pt deposition within p-type macroporous silicon forming particles within the pores. As supporting electrolyte Na_2_SO_4_is used. (b) Pt deposition into the pores in using NaCl as supporting electrolyte resulting in metal-rods. (Reproduced by permission of The Electrochemical Society [101].)

Electrodeposition of Au, Pt and Pd into macropores has been shown by Fukami *et al*. [101], whereas Pd and Pt starts growing from the pore bottom but for the deposition of Au no condition could be found to achieve this behaviour. The deposition is performed under cathodic conditions and choosing an appropriate supporting electrolyte to shift the open-circuit potential to negative values which inhibits oxidation of the silicon. Since the equilibrium potential of Au is more positive compared with Pt and Pd the rate of hole injection is faster which is a result of the greater difference between the equilibrium potential and the silicon valence band. Thus Au cannot be deposited from the bottom. In case of Pt the process begins at the pore bottom due to the high electric field strength at the tips of the pores if the proper electrolyte composition is employed (addition of NaCl). In case of using a Na_2_SO_4_ solution the nucleation begins at the pore opening. Figure 16 (taken from reference [101]) shows the comparison between these two cases of different electrolyte composition.

Cu-filling into mesoporous silicon has also been investigated concerning the value of the current density as well as in relation to the pore-length [104]. Continuous filling of the pores (diameter 40 nm) has been observed for a length of 4 µm and an applied current of –5 µA. By increasing the current to twice the value Cu has been precipitated as particles, uncontinuously. A similar result has been gained when the pore-length has been enhanced to 8 µm. These experimental examinations together with numerical simulations [104] lead to the suggestion that electrochemical filling of porous silicon with Cu depends on the mass transport of cupric ions and thus on the depth of the pores, concentration of the metal ions and the applied current.

**Figure 17:**
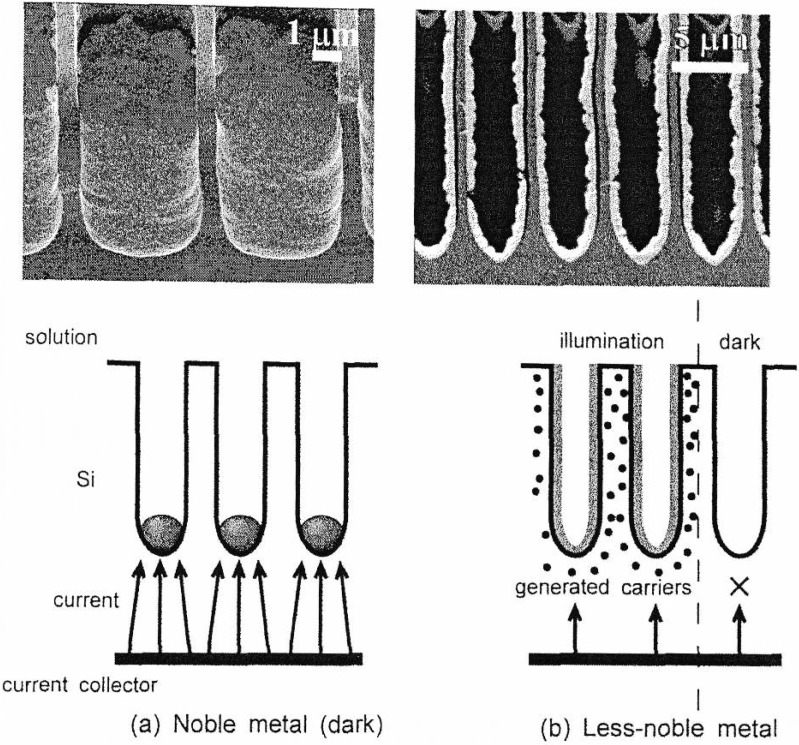
Scanning electron micrographs showing (a) the Cu-deposition within p-type macroporous silicon forming rods and (b) the deposition of Ni which covers the pore-walls. The image is taken from reference [105].

Metal deposition on silicon in general and in particular the deposition of copper, forming microrods within macroporous silicon and of nickel resulting in microtubes, covering the pore-walls of the porous silicon structure has been described by Ogata *et al*. [105]. Metal deposition forms a Schottky barrier between silicon and electrolyte when the work function of the metal is higher than the one of silicon, in case of n-type and smaller in case of p-type silicon. In case of depositing noble metals when the electron transfer *via* valence band takes place metal nucleation starts at the pore bottom. For less noble metals the occurrence of the precipitation has to be supported by illumination which permits the reduction at the pore-walls leading to the formation of metal microtubes. Figure 17 shows the formation of Cu-rods and Ni-tubes in p-type macroporous silicon, respectively.

Furthermore position-selective metal deposition on silicon by local illumination by laser beam can take place due to a local cathodic reaction caused by photo-excitation. Thus this is a promising method which enables maskless patterning of the silicon substrate. Local deposition of Ni onto porous silicon by photo-excitation of the silicon substrate, which is a method for direct metal patterning, has also been studied [106]. On the spots which are illuminated by the laser beam Ni deposition preferentially takes place due to photo-induced electrons in the conduction band which contribute to the reduction of Ni ions.

A further sophisticated process for selective metal deposition onto porous silicon is a two-step patterning process by AFM [107]. First oxide-covered silicon is engraved by scratching with a diamond tip equipped AFM and subsequently electrodeposition of Au is performed in using a standard electrochemical equipment. Another method to deposit metals on and into porous silicon is chemical vapour deposition [108] which leads to thin films on the surface but also to thin layers covering the pore-walls down to a depth of a few microns. The thickness of the covering film depends on the pore-length. It decreases with increasing depth.

## 5. Deposition of Ferromagnetic Metals

In recent years porous alumina templates formed by anodization of aluminium in an oxalic acid solution under constant voltage are the most popular, especially to produce nanowires. This material shows a periodic pore arrangement in a hexagonal manner with uniform pore distribution and high pore density. The pore diameters are generally in the range between 70 nm and 100 nm, whereas the pore depth measures a few micrometers [62]. These matrices are filled with a metal in a further electrochemical procedure. Arrays of Ni nanowires ordered in a hexagonal arrangement are investigated magnetically with respect to their arrangement [109], the dipolar coupling between the filled pores [110] and also the anisotropic behaviour [111,112].

Porous media with incorporated metals or ferromagnetic materials have a high potential for future applications and therefore are of great interest. In microelectronics silicon is the material most used. Silicon as basic material is therefore of interest for future applications, and it is favourable to produce silicon templates with pores perpendicular to the surface and of high aspect ratio. The deposition of various metals as In, Au, Cu, Fe, Ni and Co has been carried out either electrochemically or by CVD technique as described above. Anyway, a homogeneous filling of pores exhibiting a greater aspect ratio than 100 has not yet been achieved. The production of magnetic nanowires, incorporated in the porous silicon template however necessitates the homogeneous filling of the oriented channels. But also the precipitation of particles is of interest to achieve specific magnetic characteristics. These nanostructure arrays can not only be used in magneto optical recording research and technology, but they are also potential candidates for magnetic sensor applications.

The filling of porous silicon matrices with metal nanostructures is carried out electrochemically in a second process step after the anodization of the silicon wafer. Transition metals, especially ferromagnetic ones, as Ni or Co, are often used for the filling of the pores.

If porous silicon is inserted into a metal salt solution under cathodic conditions, two kinds of metal deposition are occurring. On the one hand electroless metal deposition is a possible reaction:

2M^2+^ + Si + H_2_O → 2M + SiO_2_ + 4H^+^

On the other hand electrodeposition takes place under electrical bias conditions:

M^2+^ + 2e^-^ → M

As a by-product gaseous hydrogen is formed, if the current density exceeds a certain value [113]:

2H^+^ + 2e^-^ → H_2_

The metal deposition into porous silicon which is a cathodic process reduces the metal-salt ions to metal (e.g., Ni^2+^ + 2e = Ni). Changes in the pore shape like dendritic branches as well as hydrogen evolution lead to inhomogeneities of the metal deposit, and homogeneous filling of the pores is extremely difficult to obtain. If the exchange of electrolyte is insufficient along the entire pore length, pores are blocked and homogeneous metal-filling along the whole length is inhibited. The degree of pore filling is dependent on the current density as well as on the pulse duration. This mode of application needs optimization for standardized production of tailored samples, as already applied for the specific fabrication of porous silicon templates [114]. A plausible mechanism to describe the loading of the porous matrix can be done by considering that when the cathodic reaction starts, the current density is limited by the free charge carriers supplied by silicon at the pore tips. In this tip region the charge exchange takes place and so Ni nucleates at the very end of the pores. During deposition the Ni^2+^-ions are reduced to Ni, and this process leads to decreasing concentration of the electrolyte inside the pores. Thus, the electroactive species within the pores are depleted. In consequence of the reduced electrolyte concentration within the pores the solution outside the pores is more concentrated, and therefore the mass transfer from the electrolyte sets the limiting factor of the current. So it is important that within the channels the electrolyte regenerates sufficiently during the deposition process, which is achieved in the experiments by pulsed deposition technique.

A uniform deposition of a ferromagnetic metal from the pore top to the pore tips of high aspect ratio (>100) pores is not yet achieved due to the limited mass transport and the concomitant gradient of the concentration of the depositing material along the pore depth. The uniformity of the precipitated Ni within the pores depends on the distribution of the deposition rate of Ni along the entire pore-depth which can be understood as a current distribution problem, whereas the Ni deposition rate corresponds to the ion diffusion or cathodic reduction rate. The distribution of the deposition can be determined by the mass transport along the pores and the surface kinetics of the deposition reaction [115]. Because of the small dimensions of the channels and the high aspect ratio the deposition rate is not uniform, which counteracts the homogeneous metal filling of the pores. External agitation of the electrolyte does not considerably improve the filling results in case of such high aspect ratio structures [115]. The main problem is the exhaustion of the metal-salt solution along the pore-depth. To improve the supply with metal-ions by the electrolyte within the channels the cathodic deposition process is necessary to be carried out in a pulsed way. On the other hand this provides the opportunity to tune a further parameter to affect the result of the metal-filling. Depending on the applied deposition current density and the pulse duration the spatial distribution of the precipitated metal can be influenced. Further a modification of the shape (spheres, ellipsoids, wires) of the deposited metal nanostructures within the channels is possible by varying the deposition parameters (current density between 10 mA/cm^2^ and 35 mA/cm^2^, pulse duration between 5 s and 40 s). A difficulty is that the parameters are not independent from each other.

**Figure 18:**
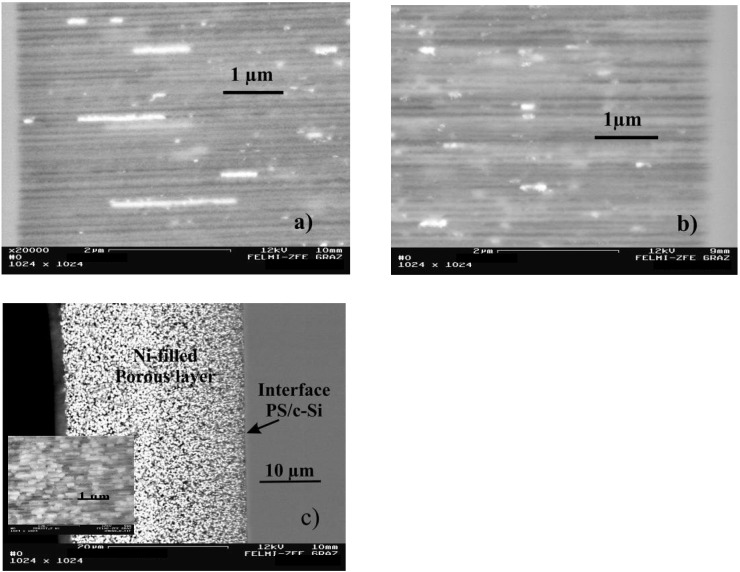
(a) Scanning electron micrograph using the back scattered electrons showing Ni-nanowires with an elongation of about 2.5 µm (current density = 40 mA/cm^2^, pulse duration = 5 s). (b) Ni-nanostructures deposited into a porous silicon matrix with a length of about 200 nm (current density = 40 mA/cm^2^, pulse duration = 20 s). (c) Back scattered electron (BSE)-image of the cross-section of an entire porous layer filled with Ni particles. The filling factor was estimated to be about 25%. Inset: Example of a zoomed area of sample 3c. A series of such images has been used to estimate the average distance of the Ni-particles to a few hundred nanometers. [114]

In case of Ni-deposition, metal wires near the pore-tips with an elongation of a few micrometers, reaching an aspect ratio of about 100 are achieved (Figure 18a). Considering the precipitated metal structures along the channels the elongation decreases up to the pore-top where the metal precipitations become more elliptical and spherical. Variation of the deposition parameters enables to influence the length of the metal-structures. For the considered specimens the diameter of the precipitations is always identical to the pore-diameter. Figure 18b shows Ni-structures of a few hundred nanometers in length embedded in channels with a diameter of 60 nm and in Figure 18c a complete filling of the porous layer with particle-like structures can be seen.

Assuming a complete filling of the channels allows to determine the filling factor only by a relation between the wire-radius *r* and the interwire separation *i* given by equation (2) [116]:

(2) $$f=\frac{2\pi r^{2}}{\sqrt{3}i^{2}}$$

### 5.1. Pore filling

This equation is related to a hexagonal closed packed pore arrangement. In case of the investigated PS-matrices the pore arrangements are in the crossover between irregular and regular arranged channels offering a four-fold symmetry (see ref. [83]). For a rough estimation of the pore-filling the above relation is used and compared by estimations carried out from magnetization measurements. The amount of Ni filled into a PS-template with an average pore-diameter and a mean pore-distance of the same value is rather high (see Figure 18c). The precipitated Ni-particles offering an average length of 200 nm are rather homogeneously distributed over the entire porous layer with an average distance of less than the particle-length estimated from a series of BSE-images (like the inset of Figure 18c). This specimen is used to estimate the filling factor of the deposited metal within the channels. On the one hand a rough approximation is carried out in using the relation between pore-area and unit cell area in a hexagonal lattice given by equation 1, being *r* the wire-radius and *i* the interwire separation. The estimated maximum filling factor, considering a complete filling of matrices exhibiting an average pore-diameter of 60 nm and a pore-distance in the same range is about 36% with respect to the whole porous sample volume. For this purpose BSE-pictures of the cross-section of the whole porous layer have been considered and analyzed in using image processing. Taking into account that the picture does not show only the top pores of the cross-section but also pores down to a certain depth, depending on the energy of the primary electrons, leads to a lower filling factor between 20% and 30%, determined from micrographs obtained from varying energies between 3 kV and 12 kV. The deposited Ni volume given in cm^3^ is approximately 3.813 (10^-6^ cm^3^). An additional estimation of the filling factor can be performed by using the corresponding magnetization data as anisotropy field H_A_ and saturation magnetization M_S_ of the deposited metal (equation 3) which are gained from magnetization measurements performed by SQUID-magnetometry and FMR-measurements:

(3) $$f=\frac{2\pi M_{S}-H_{A}}{4\pi M_{S}}$$

The anisotropy field determined from FMR-measurements is H_A_ = 1.5 kOe. M_S_ of Ni is 485 emu cm^-3^. Using these magnetic characteristics M_S_ and H_A_ of the specimens a filling factor of 25% could be determined which is in good agreement with the above estimation using equation 2 and considering the space between the deposited Ni nanostructures and the fact that not every pore is filled with exactly the same distribution of Ni.

### 5.2. Nickel deposition within porous silicon

A complete continuous filling of the pores, to achieve ultra high aspect ratio metal nanowires, is a great challenge, but the obtained results, especially the possibility of an initial metal-growth at the pore-tips, are promising for a uniform filling of the channels between pore-bottom and pore-top.

So far the Ni-deposition within the pores consists of a distribution of spherical and elongated nanostructures leading to a distribution of differently shaped precipitations. During the galvanic process this distribution can be tuned by the deposition parameters. The shape of the deposition mainly depends on the electrochemical parameters such as the applied deposition current and the pulse-duration of the current. Table 3 gives the current-parameters for a Watts electrolyte with a concentration of 0.2 M NiCl_2_ and 0.1 M NiSO_4_ and the resulting shape of the Ni-deposits.

**Table 3:** Electrochemical parameters of the galvanic process to achieve differently shaped Ni-nanostructures.

Sample	Current density [mA/cm^2^]	Pulse duration [s]	Shape, elongation [nm]
1	20	30	Conglomerate
2	40	20	Particles, 200
3	40	5	Wires , 2500

**Figure 19:**
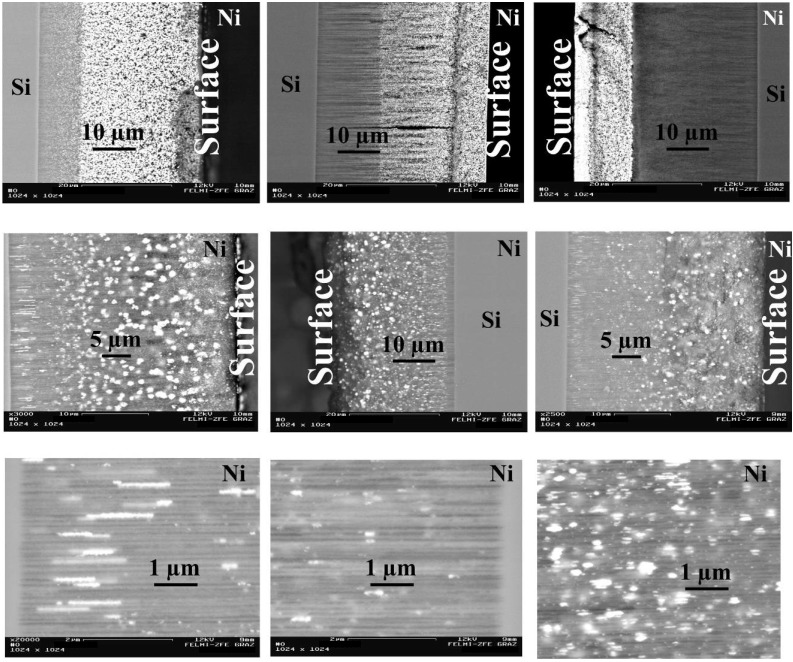
Scanning electron micrographs (BSE) showing the different arrangements of the deposited Ni-precipitations within the channels of the PS-matrices [94]. First row: the spatial distribution of the deposited metals varies between 2/3 to 1/3 of the porous layer. Second row: the porous layers are filled between surface and bottom of the pores but the shape of the precipitated Ni-structures differs. Third row: zoomed areas of row two.

Considering the precipitated Ni-structures within the channels a three-dimensional self-assembled array of ferromagnetic nanoparticles is achieved, whereas the shape and distribution along the channels can be varied by adjusting the deposition parameters as described in Table 3. Considering the top view of the samples the spatial arrangement in two dimensions is given by the pitch of the pores and can be varied by the anodization conditions (see also Section 2) but the position of the precipitations in the third dimension only depends on the deposition parameters. Such differently distributed Ni-precipitations are illustrated in Figure 19. The metal-structures not only differ in their shape but also in their spatial arrangement along the pores which means a various pitch of the aligned particles [45].

**Figure 20:**
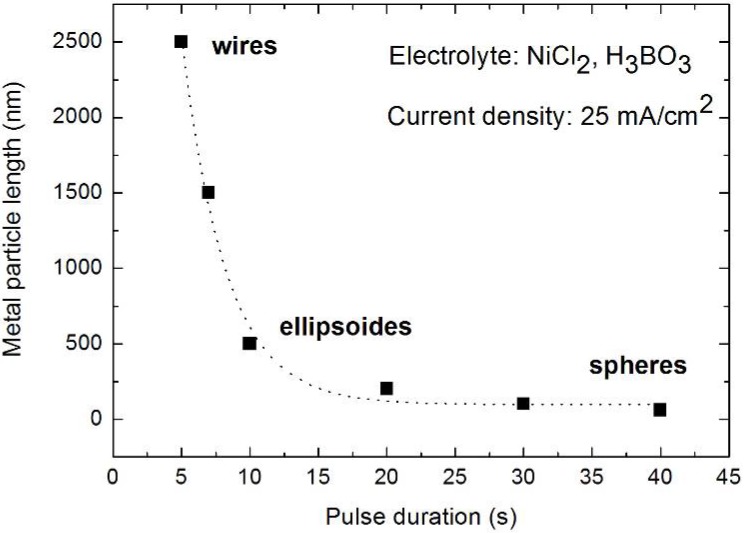
Relation between pulse duration of the applied current and elongation of the precipitated Ni-structures. The deposition time is in all cases 15 min and the applied current density was 25 mA/cm^2^. The length of the particles increases from sphere-like particles of about 60 nm up to wires of 2.5 µm. The diameter of the metal structures corresponds to the pore-diameter, which is in average 60 nm (data from [84]).

The applied pulsed deposition current with pulses ranging between 1 s and 40 s is applied to avoid the exhaustion of the electrolyte during the deposition process. If the diffusion of the metal ions is too weak the metal precipitation does not reach the pore-tips of the high aspect ratio pores and thus the filling is confined to the upper part of the porous silicon layer. A complete continuous filling of the channels exhibiting an aspect ratio of about 1,000 is difficult. In most cases the deposition is non-continuous and rather due to the formation of microcrystalline clusters [117]. The metal precipitation which is influenced by the lateral and longitudinal dimensions of the pores strongly depends on the electrolyte concentration gradient along the channels. In general, the electrochemical reaction during metal deposition preferentially takes place at the pore-tips due to the enhanced electrical field. In this case the charge transfer is the rate determining process. If the electrolyte exchange along the entire pores-length is insufficient the pores are blocked and not homogeneously filled. A decreasing concentration of the electrolyte along the pores leads to a depletion of electroactive species. Due to the gradient of electrolyte concentration between inside the pores and the electrolyte bath outside the pores the mass transfer from the electrolyte sets the limiting factor of the current. Sufficient regeneration of the electrolyte within the channels is necessary and is achieved by the pulsed deposition technique. Further reasons for inhomogeneities in the metal-filling of the pores are hydrogen evolution as a by-product during the reduction process as well as dendritic growth of the pores which results in an inhomogeneous distribution of the electrical field. The metal-filling of the entire porous silicon layer succeeded and offers a distribution of metal structures of varying size and shape. Distinct geometries of nanostructures are achieved by varying the deposition parameters like applied current density and pulse duration. The shape of deposited Ni-structures ranges between spheres (diameter due to the pore diameter), ellipsoids and elongated needle-like structures (aspect ratio up to 100) by decreasing the pulse duration from 40 s to 5 s (Figure 20).

**Figure 21:**
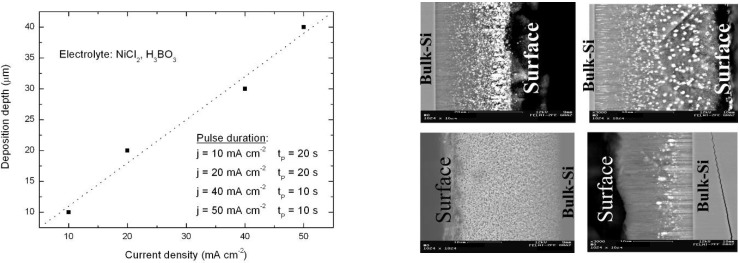
Left: Relation between current density and depth of the metal deposition in consideration of the pulse duration which has also to be adjusted [84]. Right: Micrographs gained from back scattered electrons showing the Ni-distribution within the porous silicon layers (accumulated near the surface, distribution of dispersed nanostructures over the entire porous layer, homogeneously distributed, accumulated near the pore-tips).

The spatial distribution of the deposited metal nanostructures along the pores can mainly be modified by the process parameters, as current density and coincidental modification of the pulse duration. The metal within the pores precipitates either homogeneously distributed over the entire porous layer or accumulates in a region near the surface or is mainly found in the pore-tip regions (Figure 21). The depth of the Ni-filling enhances with increasing current density. Due to the fact that the electrochemical parameters are not independent from each other the pulse duration also has to be modified over a small regime. Due to these relations between deposition parameters and metal filling nanocomposite systems with tailored magnetic properties can be fabricated.

### 5.3. Cobalt deposition within porous silicon

Co-deposition within the channels is performed in using a CoSO_4_-electrolyte with a pH-value of 4.5. The filling is also carried out in utilizing an identical electrochemical configuration by pulsed deposition technique to prevent the exhaustion of the electrolyte within the high aspect ratio pores during the loading procedure. In case of Co, so far more spherical and ellipsoidal particles have been precipitated, nevertheless the deposition within the pores is achieved down to the pore tips. For the deposition of a NiCo alloy the Ni salt solution and the Co salt solution are composed in the ratio of 2:1. The results of the deposition procedure differ for each metal with the employed electrochemical parameters. Keeping the deposition parameters constant but using different metals or compositions of metals lead also to a variety of distinct specimens of the nanocomposite.

**Figure 22:**
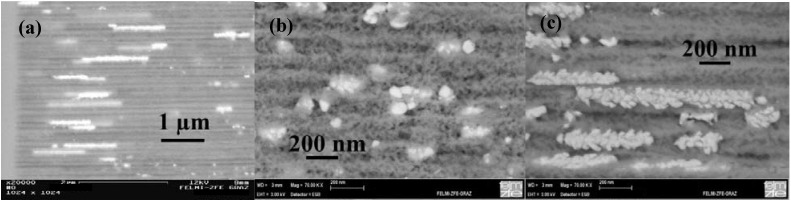
(a) Cross-sectional scanning electron micrographs obtained from the back scattered electrons of Ni-nanowires deposited within the channels of the PS matrix. The elongation of the wires exhibits about 1.5 µm and the average diameter is according to the mean pore-diameter of 55 nm [84]. (b) Precipitated Co-particles within the PS-template. The particle sizes are distributed between spheres of about 60 nm and ellipsoids with a maximum length of 150 nm [84]. (c) NiCo-nanostructures within the pores exhibiting a maximum length of 500 nm. The variation of the length of the precipitations lies between 200 nm and 500 nm [84].

Precipitation of the various metals under alike electrochemical conditions leads to nanostructures with distinct geometry (needle-like structures, ellipsoides or spherical particles) and different distributions along the pores. The results of a deposition of Ni, Co and NiCo performed under equal electrochemical conditions are shown in Figure 22. The deposited Ni-wires exhibit a length up to 2 µm and a diameter of about 55 nm. Co precipitates in ellipsoidal particles with a maximum length of about 150 nm and a diameter around 55 nm. The used NiCo composition offers nanostructures with a maximum length of 500 nm and a diameter of 55 nm. In all three cases the current density and the pulse duration of the applied deposition current had been equal (current density j = 25 mA/cm^2^, pulse duration t_p_ = 10 s).

Thus, using the same templates the geometry of Ni-precipitations can be tuned between spheres, ellipsoids and wires, in addition to their spatial distribution within the porous layer (see Figure 19 and Figure 21) in adjusting the deposition parameters especially the pulse duration and the current density of the applied current. In using the same PS-matrix and varying the process parameters Co deposits preferentially in spherical-like and ellipsoidal structures with a maximum length of about 200 nm over the entire porous layer. Up to now NiCo has been incorporated in these PS-specimens up to a maximum particle-length of about 500 nm.

### 5.4. Metal-deposition within double-sided samples

**Figure 23:**
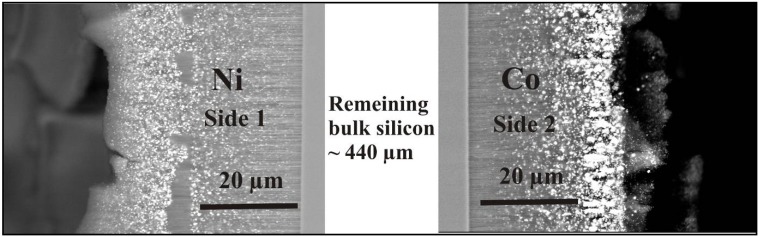
SEM-image, gained from back scattered electrons, of double-sided porous silicon with Ni deposited into the pores on one side and Co on the other side. The remaining bulk silicon between the layers is about 450 µm, the porous layers are around 30 µm each.

The deposition of metals within two porous layers is also performed by a pulsed cathodic process whereby the reduction of the metal-ions takes place alternating on each side. The frequency of the deposition is performed between 0.05 Hz and 0.2 Hz. The samples are filled with the same metals on each side or with different metals. Figure 23 shows a double-sided etched sample whereas one side is filled with Ni and the other side with Co, which is of interest concerning the magnetic behaviour, especially in case of ultrathin specimens. The deposited metals (Ni, Co) reach the bottom of the pores and form a distribution of discrete nanostructures of different size and shape along the pores.

**Figure 24:**
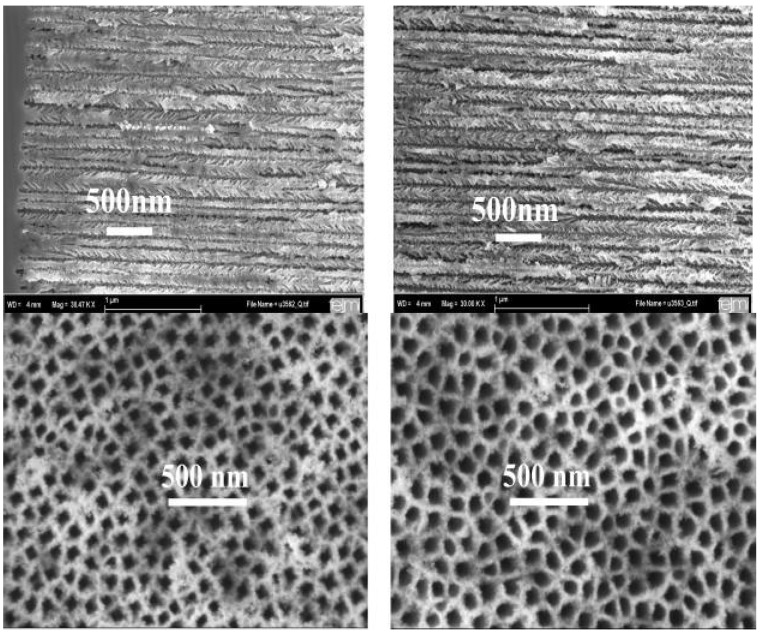
SEM-image of an enhanced region of the cross section (Figure 23) and the corresponding top view images of the two sides. The morphology of the porous structure is very similar on both sides. The average pore-diameters are around 55 nm [45].

The morphology of the porous structure on both sides of the wafer is characterized by similar dimensions (Figure 24). The average pore-diameter is 55 nm and the layer-thickness on both sides is about 30 µm.

**Figure 25:**
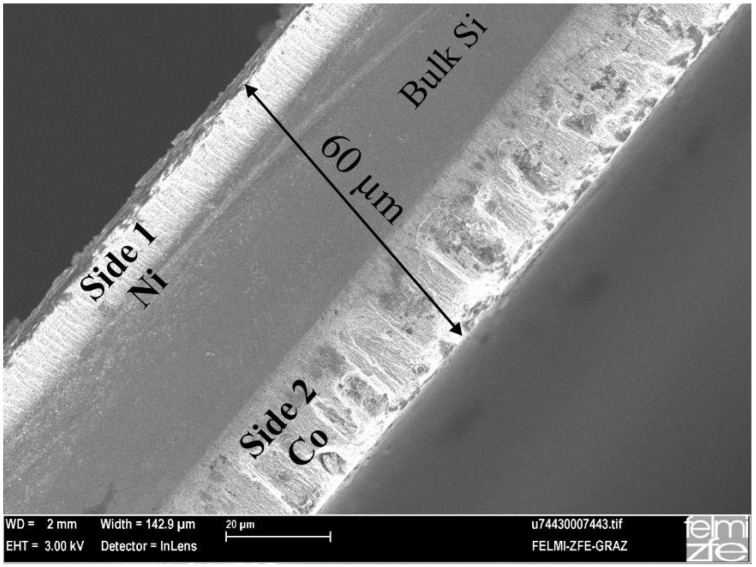
Ultrathin wafer with a thickness of 60 µm etched on both sides and filled with Ni on one side and Co on the other one. The thickness of the porous layer of side one is about 13 µm and of side two about 22 µm.

The metal-filling of ultrathin specimens is of interest to investigate the magnetic interactions between the two layers. The deposition of two different metals (Figure 25) will be especially promising for spin-injection experiments from the ferromagnetic metals into bulk-silicon [118,119]. For this aim the etching has to proceed until only a very thin (a few tens of nanometers) bulk silicon layer remains.

## 6. Magnetic Characterization

PS/metal nanocomposites, produced by electrochemically filling of porous silicon matrices with ferromagnetic metals, have been investigated magnetically. The use of porous matrices with well separated pores improves the coercivity and squareness of a hysteresis compared to magnetic thin films. Measurements of specimens with distinct magnetic properties are performed by SQUID-magnetometry. There are three possible differences between the specimens, namely the kind of metal filled into the pores, the morphology of the PS-membrane and the electrochemical process-parameters during the deposition procedure. All these parameters influence the magnetic behaviour, whereas the morphology of the porous silicon template mainly influences the dipolar coupling between adjacent pores and thus the magnetic anisotropy between easy and hard axis magnetization. Different fillings within the pores can be achieved by modifying the deposition parameters leading to nanostructures of distinct geometry and in various spatial distributions along the pores. The fabrication of the PS-membranes and the subsequent tunable metal filling is already described in detail in a previous chapter.

**Figure 26:**
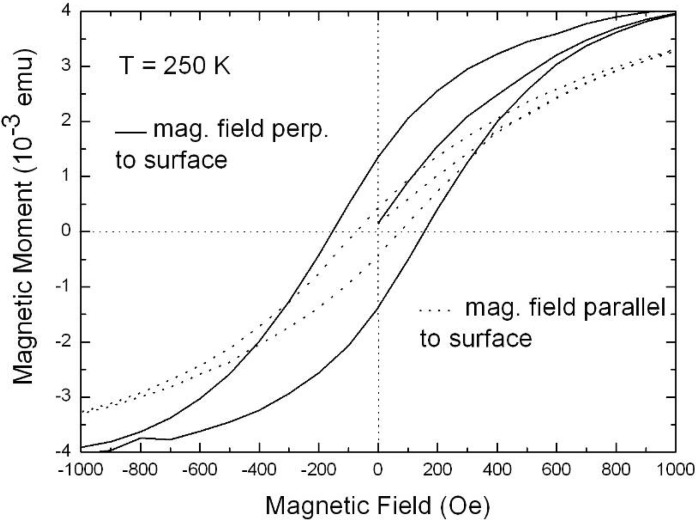
Hystereses loops of a PS-matrix filled with a large amount of Ni-wires (length of a few microns). The anisotropy between easy axis (magnetic field perpendicular to the surface) and hard axis magnetization (magnetic field parallel to the surface) is mainly caused by the shape of the deposited structures (diameter ~60 nm, aspect ratio ~80) [120].

**Figure 27:**
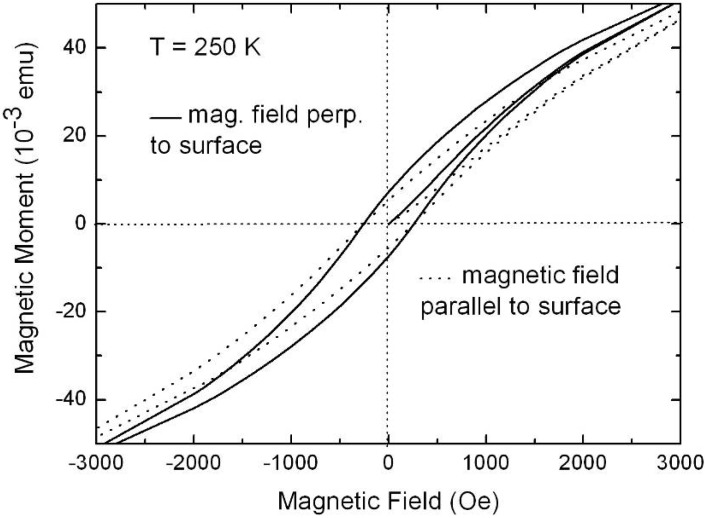
Magnetization curves of a Co-filled porous silicon sample. Co is precipitated in spherical and ellipsoidal particles reaching a maximum length of 200 nm (diameter ~60 nm). The low anisotropy between the two magnetization directions can be ascribed to the small aspect ratio of the deposits but show also that the particles within one individual pore do not strongly interact among each other [120].

**Figure 28:**
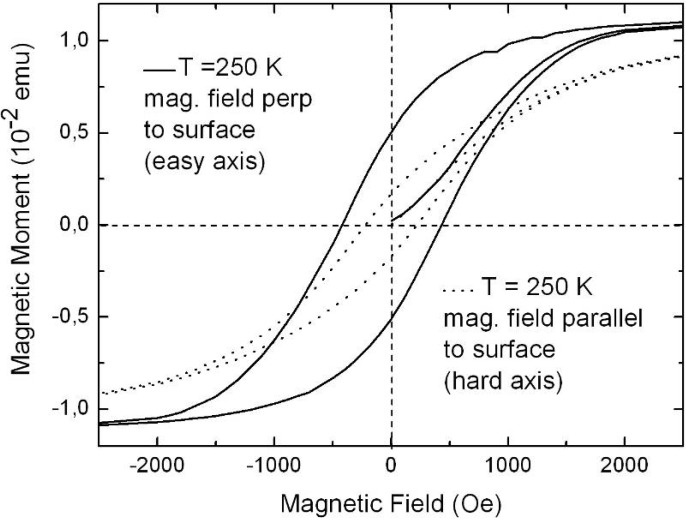
Magnetization measurements of elongated NiCo-structures (~500 nm) within PS exhibit a magnetic anisotropy of about 50% between easy axis and hard axis direction (diameter ~60 nm) [120].

Representative magnetization measurements were performed using Ni precipitated in needle-like structures of an aspect ratio up to 100, Co deposited in almost spherical particles with a maximum length of 100 nm and NiCo incorporated in granular elongated structures with a length up to 500 nm. The results are shown in Figure 26, Figure 27 and Figure 28. The measurements have been carried out in both magnetization directions, perpendicular (easy axis) as well as parallel (hard axis) to the sample surface whereas the oriented pores are grown normal to the surface. The magnetic anisotropy gives not only information about the shape of the ferromagnetic structures but also correlates with the interaction of the ferromagnetic metal nanostructures.

**Figure 29:**
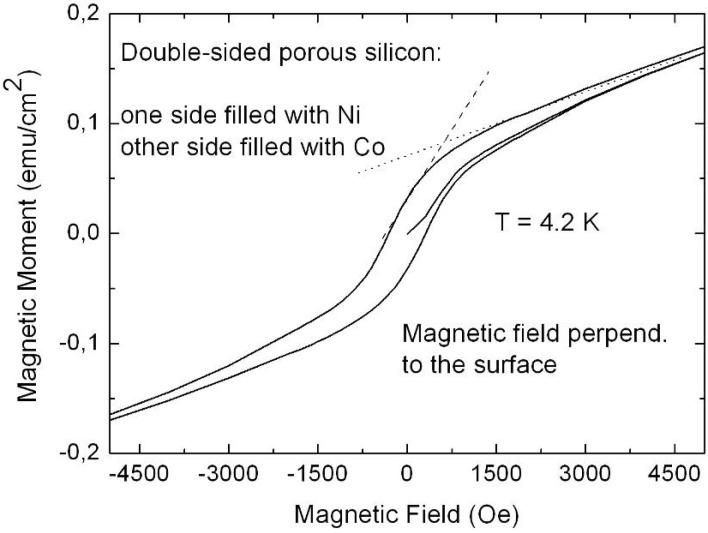
Magnetization measurements performed on a double-sided etched porous silicon sample whereas the two porous layers are filled with Ni on one side and Co on the other one. The two metals offer a distinct saturation magnetization and thus the hysteresis loop shows two different slopes.

The PS-Ni and PS-NiCo samples exhibit a magnetic anisotropy between easy axis and hard axis magnetization of about 50% which is due to the shape of the deposits. The Co filled PS specimen offers an anisotropy less than 10% due to the spheroid-like particles. Considering samples, all filled with Ni but differing in shape (wires, ellipsoids, spheres) and size of the incorporated nanostructures show also characteristic magnetic properties correlated to the geometrical characteristics (shape, size, distribution) of the deposits. The coercive field H_C_ is always smaller for embedded needle-like structures (H_C_ = 275 Oe, easy axis magnetization) than for particles (H_C_ = 520 Oe, easy axis magnetization) as shown in Figure 29 due to the stronger demagnetizing effects.

Comparing easy axis and hard axis magnetization measurements of specimens filled with the same metal show that an increase of the length of the precipitated nanostructures (60 nm up to 2.5 µm) leads to an enhancement of the magnetic anisotropy. Considering samples filled with different metals but the same size and shape of deposits show a smaller anisotropy for Co particles than for Ni-particles. This can be interpreted as a difference in the magnetic interactions among the deposits. The reason for this less interacting behaviour (e.g., oxide layer covering the particles) has still to be clarified.

A double-sided porous silicon sample, one side filled with Ni and the other one with Co shows a hysteresis loop with two different slopes caused by the different saturation magnetization of the two deposited metals. First, up to a field of 500 Oe the ferromagnetic behaviour of Ni is dominant and above the saturation of the Ni-structures the behaviour of the Co structures, which are saturated at higher fields, becomes more distinctive (Figure 29).

### 6.1. Correlation between morphology and magnetic properties

The tailored fabrication of porous silicon morphologies and the subsequent specific deposition of various transition metals within the pores of these templates result in specimens with desired tailored magnetic properties. The structural information of the nanocomposites mostly obtained by SEM- and TEM investigations is correlated with the magnetic measurements. Magnetic anisotropy gives a hint of the shape of the deposits but also information about the interactions can be gained. The obtained coercivities can also be used to obtain information about the magnetic interactions of the metal structures considering such ones of same size and shape.

The coercivity of nanowires depends mainly on contributions due to the shape, magnetocrystalline- and magnetoelastic anisotropy. Shape anisotropy affects the strength of the demagnetizing factor, and magnetocrystalline anisotropy depending on the crystal symmetry of the metal structures can be neglected due to the random crystalline orientation of the deposits. Magnetoelastic anisotropy which is usually induced by an external stress is caused on the one hand due to magnetostrictive effects but also in relation with the lattice mismatch between deposited metal and silicon skeleton. Magnetic interaction takes place due to dipolar interactions between the nanostructures, but exchange interactions cannot be excluded in case of a dense metal-structure distribution.

**Figure 30:**
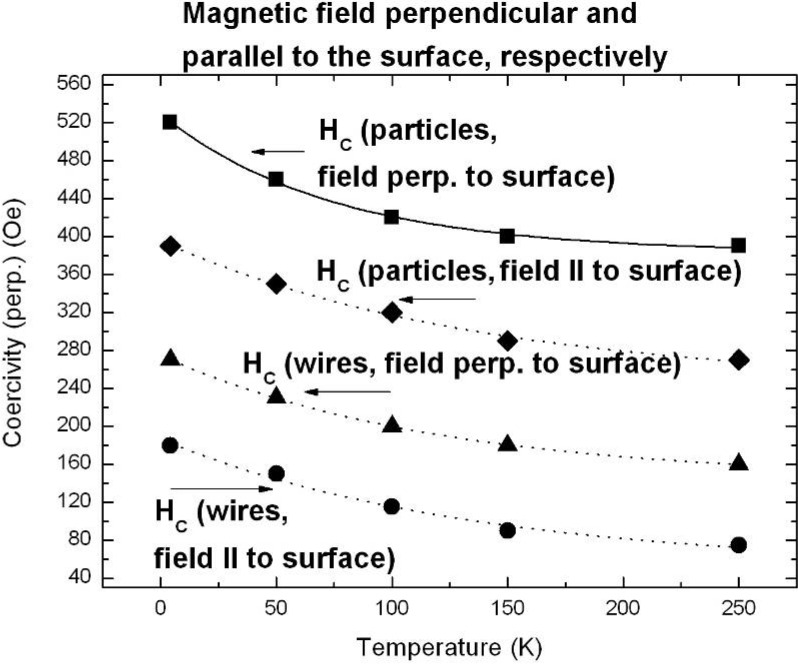
Temperature dependence of the coerciviy of two samples exhibiting the same pore-diameter and interpore spacing of the matrix (∅ ~60 nm, interpore spacing ~100 nm). One contains a large amount of embedded Ni-wires, the other one mainly Ni-particles. The measurements are performed in both directions of magnetization, with the external field applied perpendicular and parallel to the surface, respectively.

**Figure 31:**
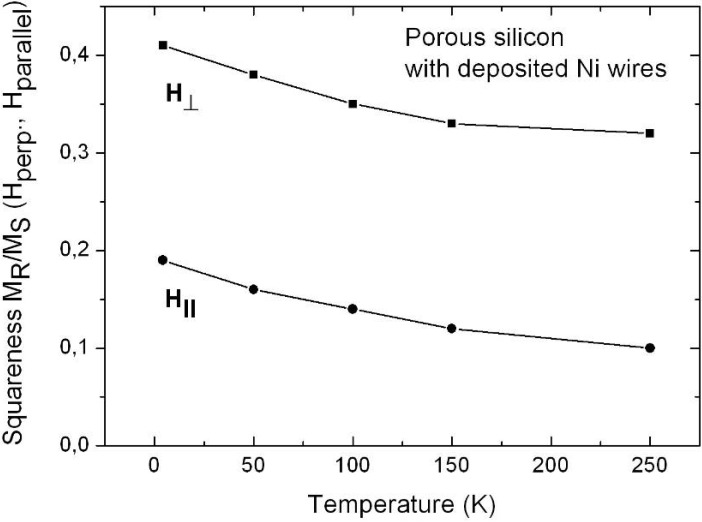
Squareness (M_R_/M_S_) depending on the temperature between 4.2 K and 250 K. For the easy axis magnetization the value decreases from 41% at 4.2 K to 32% at 250 K, whereas for the hard axis magnetization the value drops from 19% at 4.2 K to 10% at 250 K [120].

Temperature dependence of the coercivities of deposited Ni-particles and Ni-wires, respectively are shown in Figure 30. Considering embedded Ni-particles the coercive field decreases in the temperature range between 4.2 K and 250 K from a value of 520 Oe to 390 Oe (25%), considering easy axis magnetization. Samples with a large amount of Ni-wire deposition exhibit in the same temperature range and easy axis direction a decrease of the coercivity from H_C_ = 270 Oe to H_C_ = 160 Oe (40%). The corresponding squareness (magnetic remanence/saturation magnetization) of a typical PS-matrix with a large amount of Ni-wire deposition is shown in Figure 31 for a magnetic field applied perpendicular and parallel to the sample surface. For the easy axis direction the drop of the squareness exhibits a value of 9% and for the hard axis direction of also 9% [120]. The comparison for embedded Ni-wires and Ni-particles for the both magnetization directions at 4.2 K and 250 K is listed in Table 4.

**Table 4:** Squareness (M_R_/M_S_) for easy axis (⊥) and hard axis (‖) magnetization at two temperatures (4.2 K and 250 K).

	M_R_/M_S_ (4.2K, ‖) [%]	M_R_/M_S_ (250K, ‖) [%]	M_R_/M_S_ (4.2K,⊥) [%]	M_R_/M_S_ (250K,⊥) [%]
**Ni-particles**	18	13	57	50
**Ni-wires**	19	10	41	32

Magnetic remanence, especially the squareness, provides information about coupling mechanisms in samples [121]. Ferromagnetic structures with a random distribution of orientations offer a squareness of 0.5. In comparison structures with the easy axis aligned parallel to the applied magnetic field exhibit a squareness of 1, whereas with the easy axis perpendicular to the magnetic field a value of 0. A decrease of the squareness is caused by a diminution of the magnetic remanence. The smaller values determined from specimens with embedded Ni-wires compared to the ones with Ni-particles arise from demagnetizing effects that indicate stronger magnetostatic interactions between the metal nanostructures.

Porous silicon templates filled with Co-particles, exhibiting a maximum length of 200 nm, show a different temperature dependent behaviour than those samples filled with Ni-particles. Furthermore the magnetic anisotropy between easy axis and hard axis magnetization is less than 10%, considering the coercivity in these two directions. The determined coercivities, remanence and squareness for fields perpendicular and parallel to the surface, respectively are listed in Table 5. The coercive fields also decrease with increasing temperature. Between 4.2 K and 100 K the values differ less than 10% and at higher temperatures between 100 K and 250 K the coercivity drops to about half the value. The low anisotropy can be ascribed to non-interacting particles whereas the temperature dependence of the coercivities seems to originate from thermal fluctuations and blocking effects at low temperatures.

**Table 5:** Magnetic characteristics of a Co-filled PS-sample. The coercivities H_C_, remanence M_R_ and squareness (M_R_/M_S_) are determined for both magnetization directions, perpendicular and parallel to the surface [120].

T [K]	H_C,⊥_ [Oe]	H_C,‖_ [Oe]	M_R,⊥_ [emu]	(M_R_ /M_S_)_⊥_ [%]	M_R,‖_ [emu]	(M_R_ /M_S_)_‖_ [%]
**4.2 K**	500	480	0.01197	15	0.00985	12
**100 K**	470	430	0.01161	16	0.0096	13
**250 K**	260	250	0.00727	10	0.00536	7

### 6.2. Temperature dependent magnetic behaviour

**Figure 32:**
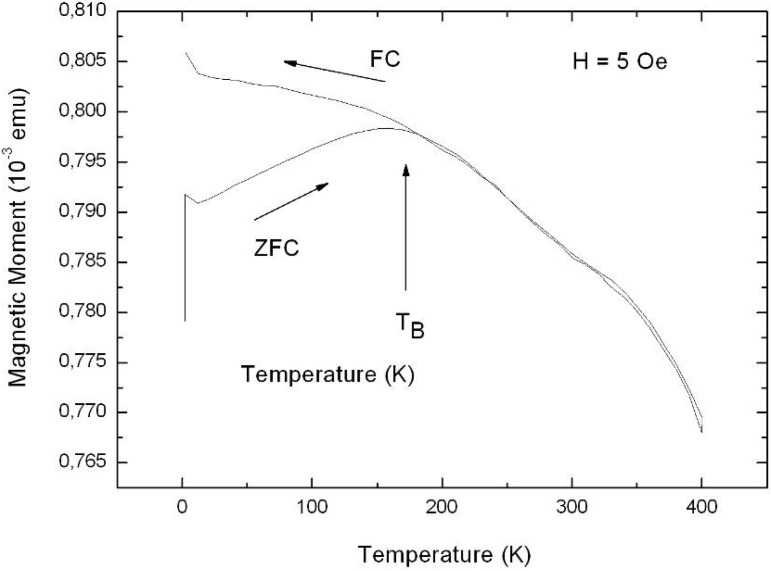
ZFC/FC measurements carried out on a NiCo-filled porous silicon template exhibiting a blocking temperature T_B_ of 180 K. The size of deposits varies between 200 nm and 500 nm [120].

**Figure 33:**
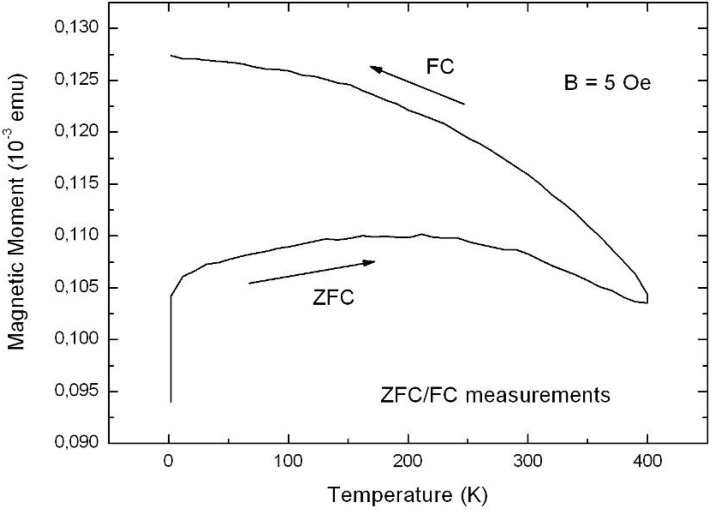
For comparison to Figure 32 ZFC/FC measurements of a PS-sample with a large amount of densely packed Ni particle deposition is investigated. The shallow increase of the ZFC-branch without the presence of a distinct peak indicates strong coupling between the Ni-structures [120].

From zero field / field cooled (ZFC/FC) measurements information about the structure can be also obtained. These measurements performed on a NiCo-filled PS-matrix at an applied magnetic field of 5 Oe are shown in Figure 32. The high blocking temperature T_B_ of 180 K indicates dipolar coupling between the metal structures. Furthermore the broad peak of the ZFC-branch not only indicates a broad size distribution of the embedded structures, but also indicates magnetic interactions between the embedded metal structures. The broad particle size distribution is confirmed by the structural SEM observation. Comparing these ZFC/FC curves with measurements of Ni-filled samples containing a large amount of densely packed Ni-particles (Figure 33) one can say that the NiCo precipitations interact less than the Ni-particles. This behaviour is caused on the one hand by less densely packed particles (estimated from magnetic measurements) and on the other hand by the use of different materials.

Considering superparamagnetic systems ZFC/FC-measurements usually show a sharp peak at low temperatures which is due to the blocking temperature. This peak shifts to higher temperatures if the particles are interacting. PS-matrices filled with a large amount of Ni-wire deposition exhibit a low decrease of the ZFC-branch with increasing temperature and also a flat FC-curve (not shown here) which both indicate demagnetizing effects due to magnetic coupling.

Magnetization measurements like hystereses loops giving information about the coercive fields, magnetic remanence and saturation of the samples and ZFC/FC-curves can be used to correlate with structural characteristics obtained from SEM-images, and give additional information about the fabricated nanocomposites.

### 6.3. Microscopic investigations delivering further information about the magnetic behaviour

**Figure 34:**
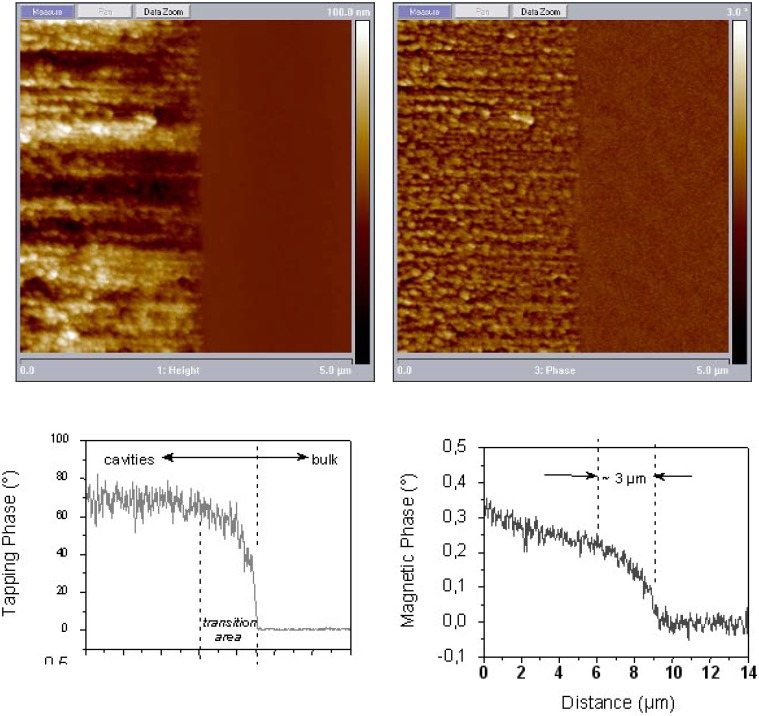
MFM-measurements performed in tapping mode (left) and magnetic mode (right) respectively showing the abrupt change between silicon and porous silicon and also the sharp difference between non-magnetic and magnetic material [122].

Further magnetic investigations of a Ni-filled porous silicon sample have been performed by magnetic force microscopy (MFM). For the investigation of porous materials the existence of a certain roughness is a drawback and complicates such measurements. First measurements have been performed on the cleaved edge of the sample near the transition between porous layer and bulk silicon [122]. Not only the topographic change between porous material and silicon has been determined, but also the magnetic signal which arises at the transition region to the porous material. Figure 34 shows the topography and the magnetic phase of the cross-section. A clear magnetic signal is observed. The MFM measurements show that the precipitated particles are magnetically aligned along the pores whereas the direction of magnetization, parallel or antiparallel to the pores, cannot be seen in case of scanning a cross sectional region. Nevertheless the magnetic alignment of the particles within the pores is in good agreement with the SQUID-measurements, mentioned above.

**Figure 35:**
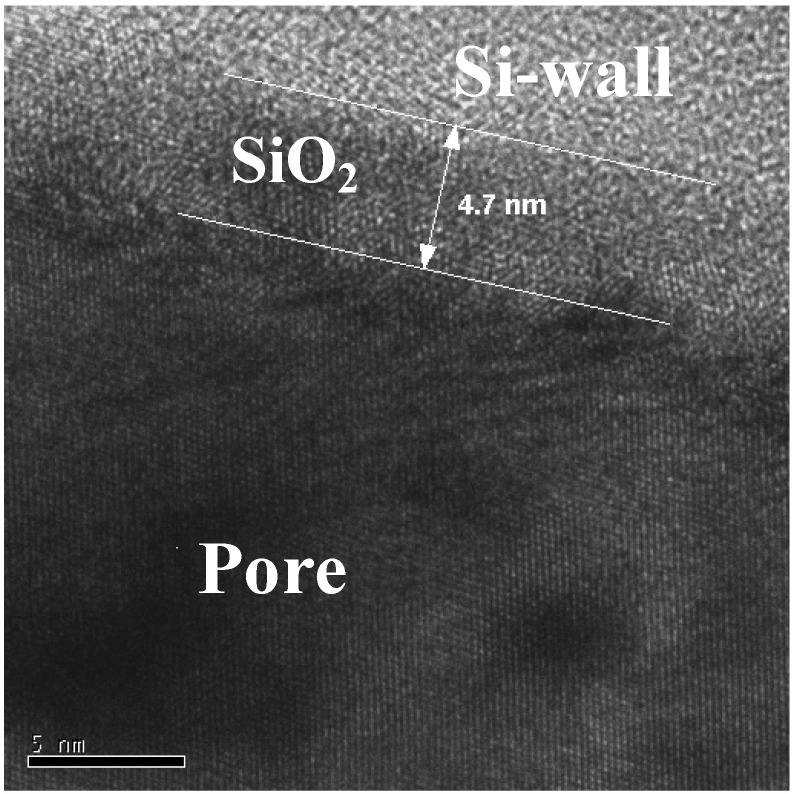
HRTEM-image of porous silicon, showing a native oxide layer of about 5 nm covering the pore-wall.

**Figure 36:**
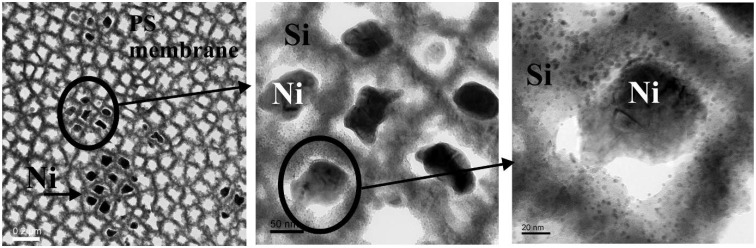
From left: TEM-image of a Ni-filled porous silicon membrane. Ni-particles can be seen inside the pores. Middle: zero-loss image of an enhanced region showing the Ni-structures within the PS-matrix. Right: HRTEM-image showing an individual Ni-particle inside a pore and the surrounding PS-skeleton.

Transmission electron microscopy (TEM) investigations of the PS/Ni interface show that the pore-walls of the porous silicon matrix are covered by a native oxide layer of about 5 nm (Figure 35). The oxidation of the pores is formed after the anodization by storing in air. FTIR-spectroscopy also shows the presence of oxide in case of aged porous silicon [123]. As-etched porous silicon samples which are hydrogen terminated show three typical absorption peaks around 2100 cm^-1^ due to Si-H_x_. PS/metal nanocomposite specimens also show an oxygen content which arises due to oxidation during the deposition process. Figure 36 shows the TEM-image of Ni-particles within the pores of a PS-membrane. Not all pores of the considered membrane are filled with a Ni-particle because the Ni-nanostructures are deposited randomly within the pores and one pore is not completely filled between pore tip and surface. Considering a certain level of the porous layer therefore not every pore contains a particle.

**Figure 37:**
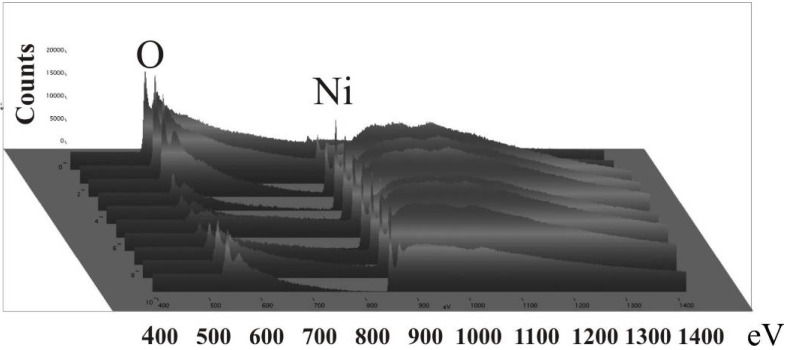
EELS (electron energy loss spectroscopy) spectrum gained from a line scan across an individual Ni-particle (Figure 36, right) within a pore shows the Ni and also oxygen. The high oxygen peak at the edge results from a mixture of SiO_2_ covering the pore wall and NiO [123].

Furthermore the preparation technique by focused ion beam (FIB) provokes the loosening of particles. Typically membranes with a thickness of about 50 µm are fabricated. The deposited metal (Ni) structures are also covered by oxide (Figure 37), which likely arises after the preparation by focused ion beam. On the other side magnetization measurements of Ni-filled samples do not show an exchange bias effect (not shown here), which means a shift of the hysteesis loop on the abscissa which generally occurs due to coupling of ferromagnetic/antiferromagnetic structures. This finding indicates that the Ni-oxide coverage of the nanostructures is not antiferromagnetic.

**Figure 38:**
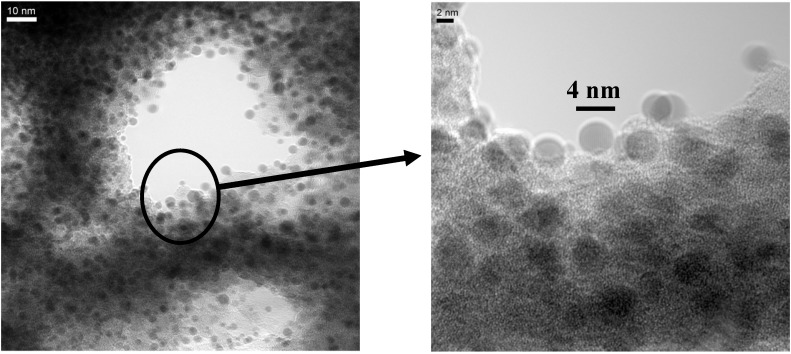
HRTEM-image showing small Ni-particles with a size between 2 nm and 5 nm covering the walls of the porous silicon skeleton. Right: enhanced region of the left image showing these small spherical Ni particles in detail.

Further investigations of the interface between porous silicon and embedded Ni-structures show that the pore-walls are covered by very small Ni-particles between 2 nm and 5 nm in size (Figure 38).

Due to the fact that the magnetocrystalline anisotropy of Ni is small, the main contribution of the anisotropy stems from the shape of the deposited metal structures. On average the deposited Ni-structures of the considered sample offer a diameter of about 50 nm and a length of about 150 nm. The magnetic anisotropy of an individual nanowire is dominated by shape anisotropy (1/2 μ_0_M_S_ ≈ 10^5^ J/m^3^) [110]. In case of deposited Ni-particles coupling between the particles is expected due to the large anisotropy between the two magnetization directions. So it is reasonable that the precipitated Ni-particles dipolarly couple within one pore leading to a quasi “magnetic chain” which enhances the anisotropy. This coupling between the “bigger” Ni-structures seems to be mediated by the small Ni particles which also dipolar interact.

## 7. Porous Silicon/Fe_3_O_4_-Nanocomposite

The fabrication of isolated magnetic nanoparticles is difficult to achieve because the large surface areas oxidize easily when using metals and they tend to agglomerate due to the magnetic interactions. A controlled passivation of the particles can be carried out, but this can also lead to interactions between the metal core and the passivating materials. Magnetic iron oxide such as magnetite has the advantage of being more stable. Recently, a preparation method has been reported based on the decomposition at high temperature of an organic precursor in the presence of oleic acid, which leads to a monodisperse size-distribution of nanoparticles isolated by an oleic acid layer of around 2 nm [124,125,126].

The combination of nanostructured silicon and magnetite leads not only to interesting magnetic properties of the nanocomposite system, but is also a good candidate for applications in biomedicine because both are biocompatible in biological systems due to their low toxicity and biodegradability [127,128].

**Figure 39:**
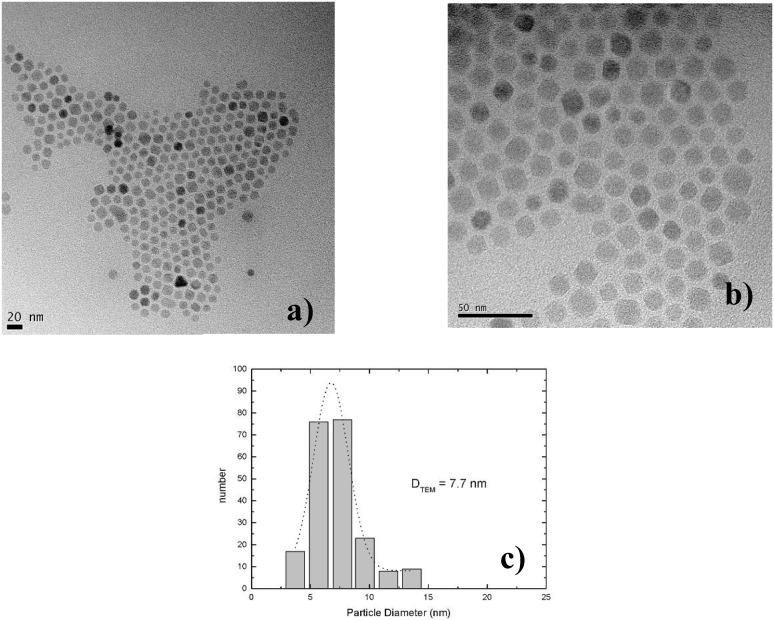
(a) TEM-image of the quite monodisperse magnetite nanoparticles, (b) enhanced region. (c) particle size distribution giving an average diameter of 7.7 nm which is gained from the element (Fe) distribution of the sample due to better contrast (reproduced by permission of The Electrochemical Society [131]).

Magnetite nanoparticles of an average size of 8 nm have been prepared by high temperature decomposition of iron organic precursors at the CSIC Institute of Material Science in Madrid, Spain. These particles have been synthesized using iron acetylacetonate as precursor and phenyl ether as solvent. A mixture of 0.71 g of Fe(acac)_3_ (2 mmol), 2.38 g of 1,2-hexadecanediol (10 mmol), 1.69 g of oleic acid (6 mmol), 1.60 g of oleylamine (6 mmol) and 20 mL of trioctylamine have been added to a three-neck flask. Then, the mixture reaction has been heated under mechanical stirring and a flow of nitrogen gas until a temperature of 200 ºC has been reached. This temperature has been kept constant for 120 min and then the solution has been heated to reflux (369 ºC) for 30 min in a nitrogen atmosphere. Finally, the solution has been cooled down to room temperature. The powder was obtained by precipitation with ethanol, collected with a magnet and finally dried under nitrogen flow. A stable suspension of the powder could be obtained when nanoparticles have been mixed with 20 mL of hexane and 0.05 mL of oleic acid and sonicated for a time period of 5 minutes. The achieved particles show a quite monodisperse size-distribution (Figure 39c) of 9 nm obtained from TEM-images (Figure 39a, b) with a deviation of ±1 nm.

Magnetite nanoparticles have been deposited onto hydroxyl functionalized porous silicon samples resulting in a self-organizing dendrite-like arrangement on the surface which offers a new composite material [129]. The used magnetite nanoparticles which are superparamagnetic show dendrite-like formation caused by a diffusion limited aggregation model [130].

### Influence of the porous silicon matrix

In our work we infiltrated the suspension of magnetite nanoparticles in hexane solution into the pores of the mesoporous silicon matrix. This immersion has been carried out under defined conditions (e.g., concentration of the solution, temperature). The infiltration time has been 30 min. The Fe_3_O_4_-particles used for infiltration into the PS-template coated with oleic acid in a hexane solution exhibit an average diameter of 8 nm and only a distance of a few nanometers (~3 nm) between them (Figure 39). The narrow size-distribution and the superparamagnetic behaviour at room temperature are interesting features of these nanoparticles.

A comparison of a porous silicon template and a PS/Fe_3_O_4_-sample has been studied by FTIR-spectroscopy [131]. In addition to the Si-H stretching modes of the porous silicon, occurring in both samples, C-O stretching modes have been identified at 1,530 cm^-1^ and 1,625 cm^-1^, which is in agreement with IR-investigations of magnetite nanoparticles [132]. The Fe-O modes at 430 cm^-1^ and 610 cm^-1^ could not be found because of the absorption edge of the silicon substrate at 1,200 cm^-1^. Additional peaks around 2,260 cm^-1^ indicate oxidation of the PS-matrix (H-Si(O_3_)-modes).

**Figure 40:**
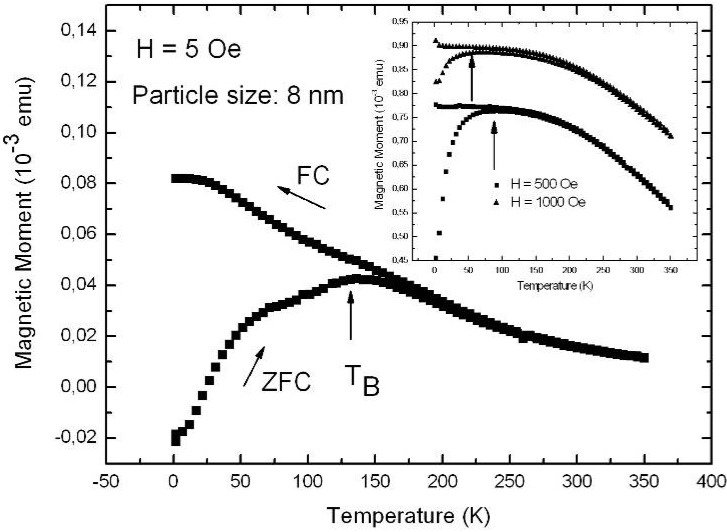
ZFC/FC measurements carried out at an applied magnetic field of 5 Oe and in a temperature range between 4.2 K and 360 K. The high blocking temperature (maximum peak of the ZFC-branch) at 135 K indicates dipolar coupling between the particles. Inset: Shift of T_B_ towards lower temperatures with increasing applied magnetic field (reproduced by permission of The Electrochemical Society [131]).

The achieved magnetic system with porous silicon acting as substrate and infiltrated magnetite nanoparticles leads to a composite material showing a ferromagnetic behaviour at low temperatures (T < T_B_) and superparamagnetism at higher temperatures (T > T_B_). This transition temperature can be influenced by the particle size but also by the distance between the particles. The superparamagnetic behaviour of the magnetite/porous silicon system above a blocking temperature T_B_ is shown by temperature dependent magnetization measurements. Zero field cooled (ZFC)/field cooled (FC) investigations performed at an applied field of 5 Oe show a rather high blocking temperature T_B_ at 135 K which indicates magnetic interactions between the particles (Figure 40). Furthermore, a shift of the blocking temperature to lower temperatures with higher applied fields is observed (inset Figure 40). This behaviour of superparamagnetic particles is known to be proportional to H^2/3^ at high fields and proportional to H^2^ for lower fields [133]. The blocking temperatures determined for different applied magnetic fields are summarized in Table 6.

**Table 6:** Blocking temperatures for magnetic fields between 5 Oe and 1,000 Oe.

**H [Oe]**	5	500	1,000
**T_B_ [K]**	135	75	55

Considering the ZFC/FC measurements one recognizes that the splitting temperature between the ZFC and the FC-branch differs from the blocking temperature which coincides with the maximum of the ZFC-curve. Such a behavior is observed in randomly dipolar coupled nanomagnet-systems. Also the width of the peak of the ZFC-branch can be attributed to dipolar coupling of the nanoparticles since the distribution of the particle-size is quite monodisperse proven by TEM-images.

Using the thermal energy:

(4) $$25k_{B}T_{B}=KV{\left(1-\frac{\mu_{0}M_{S}H_{C}}{2K}\right)}^{2}$$

with:

K … anisotropy constant
V… volume of the particle
M_S_ … saturation magnetization
H_C_ … coercive field

the critical diameter D_B_ for blocking at the threshold field H_C_ can be derived as:

(5) $$D_{B}={\left(\frac{25k_{B}T_{B}}{\alpha K}\right)}^{1/3}$$

where α … shape factor (= 0.5236 for a sphere).

This leads to estimated values of D_B_ of 20 nm, which is far from the diameter of the incorporated magnetite nanoparticles exhibiting an average size of 9 nm. Consequently it can be said that the particles of the investigated samples are superparamagnetic above a quite high blocking temperature of 135 K due to the presence of dipolar interactions.

**Figure 41:**
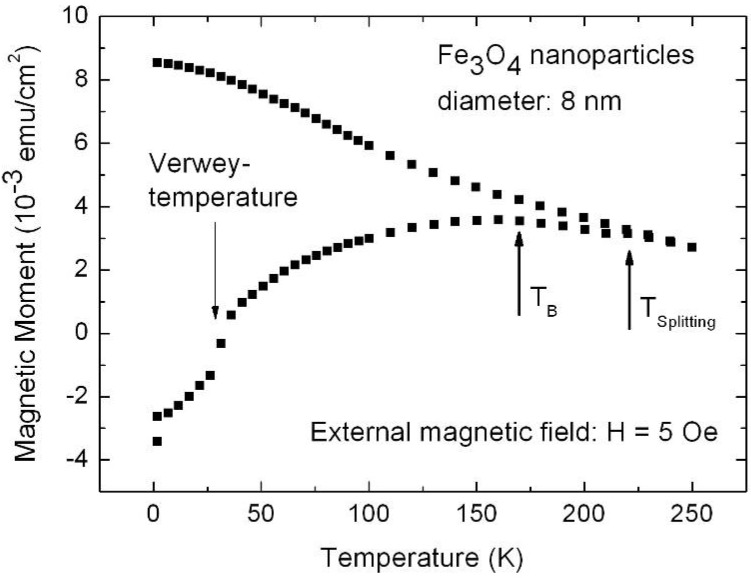
Temperature dependent magnetization measurements of Fe_3_O_4_-nanoparticles exhibiting a blocking temperature at 160 K and a bifurcation of the two branches (ZFC/FC) at 230 K (reproduced by permission of The Electrochemical Society [131]).

Considering the magnetization curve of magnetite nanoparticles (size 9 nm) without PS-matrix one sees that the blocking temperature is 160 K and the bifurcation of the ZFC and FC branches takes place at 230 K (Figure 41).

These temperatures are higher than in case of magnetite nanoparticles embedded in PS. Given that in both cases the same particles are used, a reduction in the magnetic interaction seems to take place when the magnetite nanoparticles are incorporated within the PS-matrix, which could be caused by the morphology of the PS, exhibiting a pore-distance of about 60 nm. Thus the Fe_3_O_4_-nanoparticles can only interact in one direction, along the pores. Moreover, due to the presence of oleic acid coating of the particles, exchange interaction is discarded and the interaction is mainly dipolar.

It should be noted that a turn of inflexion is observed in the ZFC curve for magnetite nanoparticles as synthesized and dispersed in the silicon matrix below 50 K which could be assigned to the Verwey transition [134].

**Figure 42:**
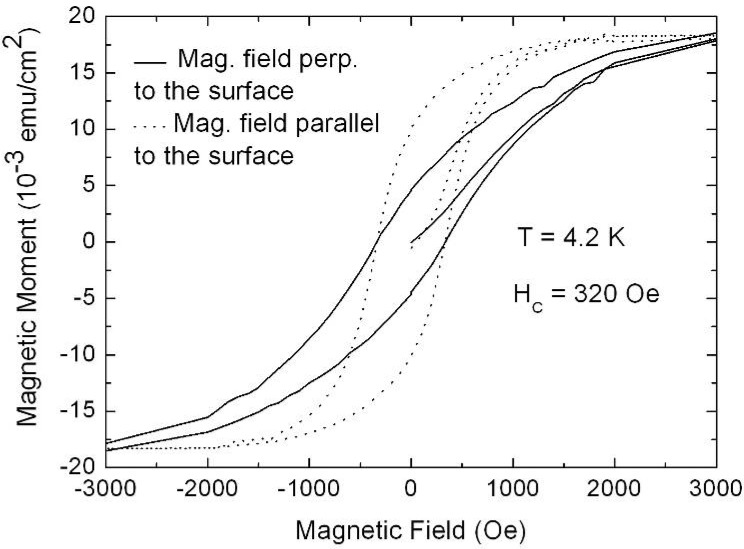
Magnetization measurements of a magnetite solution covered silicon wafer performed with the magnetic field applied perpendicular to the surface (full line) and parallel to the surface (dotted line), respectively. The obtained anisotropy is alike to the one of a magnetic film affirming that the Fe_3_O_4_-particles which form a thin layer interact dipolarly (reproduced by permission of The Electrochemical Society [131]).

Hystereses loops of a bare silicon wafer offering a surface covered with a suspension of Fe_3_O_4_-nanoparticles and a porous silicon matrix with magnetite-nanoparticles impregnated into the PS-layer are measured at T = 4.2 K. The magnetic field is applied perpendicular and parallel to the sample surface, respectively. The silicon wafer having the surface covered with the magnetite-solution shows a magnetic anisotropy between the two magnetization directions (Figure 42) which is alike the behaviour of a magnetic thin film due to the dipolar coupling of the nanoparticles.

**Figure 43:**
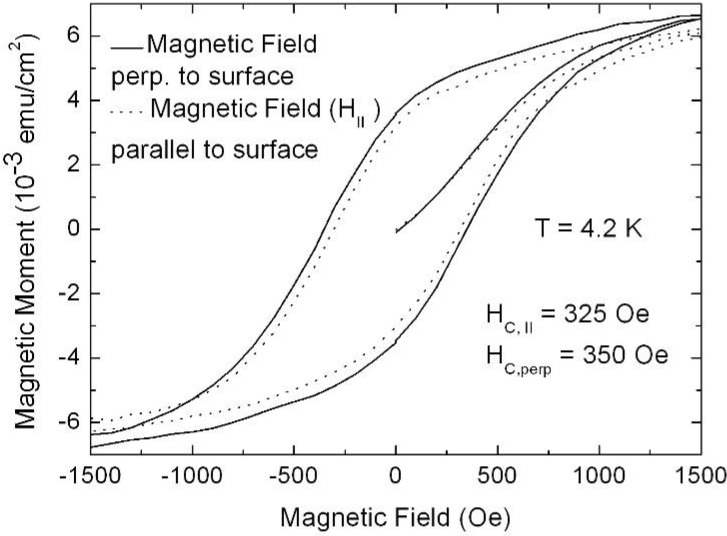
Hystereses loops of a PS-matrix impregnated with a magnetite-solution. The measurements have been carried out for easy axis and hard axis magnetization, perpendicular (full line) and parallel (dotted line) to the sample surface. The small anisotropy is mainly caused by the morphology of the used PS-template leading to predetermined stronger coupling of particles situated along one pore (reproduced by permission of The Electrochemical Society [131]).

Considering the hystereses loops of a porous silicon specimen infiltrated with the same magnetite-solution the magnetic anisotropy between the two magnetization directions is weak (Figure 43), but it differs drastically from the magnetization curve of the surface covered bare silicon wafer. The coercivities obtained for the two different magnetization directions vary a little which shows that the particles are mainly incorporated within the pores and they do not accumulate on the surface of the PS-template. The dipolar coupling between particles within one pore is stronger than for particles between adjacent pores. The average distance between the particles within one pore is about 3 nm (caused by the oleic acid coating), whereas the mean distance between neighbouring pores is 40 nm.

Magnetite is investigated nowadays because of its promising applications in nanomedicine for the location and diagnosis of tumors. Luminescent porous silicon, exploiting the light emitting properties, has been utilized to image cells *in vitro* and organs *in vivo* [55]. Due to the biodegradability and biocompatibility of porous silicon [128] a combination of these two materials could be a promising candidate for medical *in vivo* applications, especially drug delivery within a magnetic field and retarded release of tumor-attacking drugs to the affected cell. The magnetic properties of the nanoparticle/PS system are of interest due to the magnetic phase transition controlled by the strength of the magnetic interaction, which is determined by the distance between the particles and the direction, given by the matrix. Non-interacting superparamagnetic magnetite nanoparticles offer a blocking temperature below 10 K, whereas strongly interacting particles can lead to a blocking even at room temperature.

## 8. Some Potential Uses of Porous Silicon

The biological applicability of porous silicon [135], well known for many years, plays a key-role in porous silicon investigations and has been sophisticated in numerous ways to render this material deployable in various utilizations. One requirement for biocompatible materials is the hydroxyapatite growth on the surface by exposing to body fluids which is also of importance for creating new materials for scaffolds and bone substitutes. Biodegradability and bioactivity of porous silicon play an opposing role and it has been found that its oxidation can improve the bioactivity due to the reducing dissolution. Oxidized porous silicon is more stable in simulated body fluids and also crystalline hydroxyapatite can be deposited after exposing 15–30 days to these fluids [13].

The use of a composite consisting of porous silicon with high porosity and a biodegradable poly(ε-caprolactone (PCL) polymer which has been used for engineering scaffolds for skin, bone, *etc.* was investigated with respect to calcification upon exposure to simulated body fluids. The use of silicon for tissue engineering is important due to the stimulation of calcium phosphate formation which is required for bone formation [54]. The therapeutic relevance of this porous silicon/PCL composite material has been shown due to the profileration of mouse stromal cells which differenciate into osteoblasts under certain conditions [54].

Furthermore porous silicon, due to its bio-behaviour and low toxicity, is an attractive material for controlled drug delivery. The attachment of molecular payloads into porous silicon can be divided into covalent attachment of biomolecules or drugs, physical trapping by oxidation of the porous silicon matrix and adsorption which is an ion exchange holding molecules more weakly [38]. The incorporation of anti-cancer therapeutics, analgesics, proteins and peptides, as well as the use of porous silicon as dietary supplement, has been taken into consideration. Drug delivery with porous silicon is under discussion in employing particles, films, chip implants and composite materials, whereas microparticles are under great investigation because they are compatible to existing drug delivery concepts [38]. Exploration of porous silicon for utilization in cancer treatment, especially for brachytherapy has been enforced by pSiMedica Inc. [136]. Furthermore percutaneous implants of porous silicon particles loaded with a radioactive isotope have been applied to tumor radiation. The porous silicon hydrolyzes within the body to silicic acid and thus the device does not remain in the body. The concentration of silicid acid is low enough to exclude toxicity.

The combination of porous silicon with magnetic particles, namely Fe_3_O_4_, and additional loading with a molecular payload is of interest for controlled transport in applying an external magnetic field. The loaded molecules (enzymes) can be transported and subsequently released in an appropriate solution [137]. The fabrication of a porous silicon double-layer of different pore-size is used for loading with magnetite nanoparticles and a small amount of liquid. The samples are heated within an oscillating magnetic field, which is enabled by the superparamagnetic magnetite nanoparticles (~10 nm in size) [138].

One big issue of today’s research in the field of magnetic materials is still the integration of both ferromagnetic and semiconducting systems at one material level, whereas a silicon based substrate is appropriate for further integration into microtechnological processes. One way to reach this aim is the doping of semiconductor materials with transition metals resulting in so called ferromagnetic semiconductors. Up to now such systems do not operate at room temperature. Therefore the presented nanocomposite material, which is fabricated by self-assembly, offers ferromagnetic nanostructure arrays in a semiconductor material. Merging electronic properties of a semiconductor with the nanomagnetism of the embedded metal structures results in a hybrid system offering ferromagnetic properties at room temperature. To synthesize large area arrays of periodic magnetic nanostructures is a key trend in today’s nanotechnology. The introduced system, a nanocomposite of metal particles/wires embedded in a silicon matrix, offers a quasi-regular pore arrangement obtained by self-assembly with metal precipitations quite homogeneously distributed over the entire porous layer. This nanoscopic composite material exhibits magnetic properties which are not only of interest in basic research, but the system is also a promising candidate for future applications. The development of low-dimensional magnetic composites based on silicon will offer progress in spintronic which is dependent on the availability of magnetic semiconductors working at room temperature.

The combination of semiconductor electronics and spintronics, which means to use the spin of the electrical carriers to achieve similar purposes as with the charge used in today’s electronics, is one way towards achieving spin-based silicon devices. One of the first steps will be to gain sufficient spin injection into silicon because so far spin polarized currents have been mainly observed in GaAs. The work by Jonker and coworkers [119] demonstrates a way to detect spin injection from a Fe-film through an Al_2_O_3_-tunnel barrier into a silicon (100) n-i-p doped heterostructure for the first time. The detection has been carried out by the observation of the polarized electroluminescence. Fundamental properties of silicon such as smaller spin-relaxation than in GaAs, inversion symmetry of the crystal structure, absence of a nuclear spin, suppressing hyperfine interactions make silicon an adequate material for the combination of spin and electronic properties. The spin-injection can in general be done through a ferromagnet/Schottky contact, in using an oxide tunnel barrier between the ferromagnetic metal and the semiconductor or in using doped silicon as spin aligner. To employ the presented system for spin-injection the concept with the oxide tunnel barrier would be adequate. But prior to such experiments a sophisticated silicon/SiO_2_/ferromagnet structure has to be fabricated, especially a well defined grown oxide layer within the pores is necessary. Silicide formation at the interface has to be avoided because ferromagnetic atoms in the silicide phase exhibit a magnetic moment which is different to the one in the pure ferromagnetic metal. Such atoms are spinflip scattering centres and decrease the efficiency of the spin-injection.

## 9. Conclusions

Porous silicon is a versatile material which can be employed in many fields of nano-science research, but also for a great number of applications. Due to the various natures of its morphology this material can be utilized as host for loading with different molecules (gas, biological) or particles (e.g., Fe_3_O_4_). The morphology can be tuned over a broad range (2 nm up to a few microns) by modification of the anodization conditions as well as of the doping density and type of doping of the silicon wafer. Properties which occur due to the nanostructuring of bulk silicon, for example luminescence, dependence of the refractive index on the porosity, biodegradability and bioactivity renders porous silicon a material which can be exploited in optics, sensor technology, biomedicine and many more. Especially the sensing and biomedical applications (gas-, biosensors, tissue engineering, controlled drug delivery) have been emerging in recent years. The filling of porous silicon with a ferromagnetic material (Ni, Co, Fe_3_O_4_) is performed in using structures with pores grown perpendicular to the surface and separated from each other. In applying convenient electrochemical parameters a quasi regular pore-arrangement exhibiting four-fold symmetry can be achieved. Such metal filled templates exhibiting a three-dimensional array of magnetic nanostructures are promising systems, of interest for basic research due to tunable magnetic characteristics which are dependent on the filling conditions of the pores. The deposited metal structures can be varied in their spatial distribution along the pores and also in their geometry and thus samples with desired magnetic properties as coercivity, magnetic remanence and magnetic anisotropy can be fabricated. Ferromagnet/silicon composites which work at room temperature are of considerable interest especially concerning the compatibility in microelectronics. 
